# PAPγ associates with PAXT nuclear exosome to control the abundance of PROMPT ncRNAs

**DOI:** 10.1038/s41467-023-42620-9

**Published:** 2023-10-24

**Authors:** Xavier Contreras, David Depierre, Charbel Akkawi, Marina Srbic, Marion Helsmoortel, Maguelone Nogaret, Matthieu LeHars, Kader Salifou, Alexandre Heurteau, Olivier Cuvier, Rosemary Kiernan

**Affiliations:** 1 CNRS-UMR 9002, Institute of Human Genetics (IGH)/University of Montpellier, Gene Regulation Lab, 34396 Montpellier, France; 2 Center of Integrative Biology (CBI-CNRS), Molecular, Cellular and Developmental Biology (MCD Unit), University of Toulouse, 31000 Toulouse, France

**Keywords:** Chromatin, RNA

## Abstract

Pervasive transcription of the human genome generates an abundance of RNAs that must be processed and degraded. The nuclear RNA exosome is the main RNA degradation machinery in the nucleus. However, nuclear exosome must be recruited to its substrates by targeting complexes, such as NEXT or PAXT. By proteomic analysis, we identify additional subunits of PAXT, including many orthologs of MTREC found in *S. pombe*. In particular, we show that polyA polymerase gamma (PAPγ) associates with PAXT. Genome-wide mapping of the binding sites of ZFC3H1, RBM27 and PAPγ shows that PAXT is recruited to the TSS of hundreds of genes. Loss of ZFC3H1 abolishes recruitment of PAXT subunits including PAPγ to TSSs and concomitantly increases the abundance of PROMPTs at the same sites. Moreover, PAPγ, as well as MTR4 and ZFC3H1, is implicated in the polyadenylation of PROMPTs. Our results thus provide key insights into the direct targeting of PROMPT ncRNAs by PAXT at their genomic sites.

## Abstract (web summary)

Pervasive transcription of the human genome generates an abundance of RNAs that must be processed and degraded by the nuclear RNA exosome. Here the authors show that polyA polymerase gamma (PAPγ) associates with PAXT providing key insights into the direct targeting of PROMPT ncRNAs by PAXT at their genomic sites.

## Introduction

Transcription is an essential process that allows the production of RNA from the DNA genome. However, cryptic transcription, including antisense transcription, leads to the production of many transcripts that must be degraded^1–3^. Nuclear exosome is the main machinery involved in processing and degrading these cryptic transcripts. One such type of transcript that is prominent and widely studied are promoter upstream transcripts (PROMPTs), also known as upstream antisense RNAs (uaRNAs)^4^.

PROMPTs are short transcripts of about 500-2000 nucleotides in length that initiate upstream and antisense from the TSS of genes. PROMPTs must be eliminated for normal cell function. Indeed, inhibiting the degradation of PROMPTs can lead to deleterious side effects such as the inhibition of translation of mRNAs in the cytoplasm^4^. However, recent data have shown that PROMPTs are required for the transcriptional activation of estrogen-responsive genes through activation of 7SK-P-TEFb complex^5^ suggesting that the abundance of PROMPTs should be tightly regulated. Degradation of PROMPTs is carried out by the nuclear RNA exosome, a highly conserved 3′–5′ ribonucleolytic complex^6–9^. The nuclear exosome consists of 9 core subunits that form a barrel-like structure with a central channel^10,11^. The catalytic activity is conferred by association with 3′−5′ ribonucleases, Rrp6, which possesses distributive activity, and Dis3, a processive exo- and endonuclease^12–17^. Nuclear RNA exosome also associates with MTR4 (SKIV2L2), a 3′−5′ DExH-box RNA helicase that promotes unwinding and degradation of structured RNA substrates^18–20^.

Nuclear exosome utilizes adaptor complexes in order to target RNAs for degradation. The nuclear exosome targeting (NEXT) complex consists of MTR4 together with the zinc-finger containing protein, ZCCHC8, and an RNA-binding protein, RBM7. NEXT targets primarily short, mono-exonic RNAs, such as enhancer RNAs (eRNAs) and PROMPTs/uaRNAs, as well as long non-coding RNAs (lncRNAs), without a requirement for polyadenylation^21,22^. A second exosome targeting complex, consisting of MTR4 together with the zinc-finger protein ZFC3H1, is known as the polysome protector complex (PPC) or the polyA-tail exosome targeting (PAXT) connection^4,23^. PPC/PAXT consists of MTR4, ZFC3H1 zinc-finger protein, ZC3H3, RBM26/27 together with polyA binding protein, PABPN1^23–25^. The presence of PABPN1 confers a preference for polyadenylated RNAs as targets of PAXT. Indeed, PAXT and NEXT share many target RNAs, including PROMPTs/uaRNAs, eRNAs and prematurely terminated sense transcripts, although targeting likely occurs at different stages of maturation^4,23^. PAXT subunits co-localize with pA(+) RNA foci, whose formation depends on ZFC3H1^24^.

While the addition of a poly(A) tail is essential for normal mRNA biogenesis, polyadenylation can also stimulate the degradation of aberrant mRNAs and certain ncRNAs, including PROMPTs^26–30^. The canonical mammalian poly(A) polymerases, PAPα and PAPγ, catalyze template-independent polyA extension of the 3′ end of RNA^31–33^. PAPα and PAPγ have similar organization of structural and functional domains^31–34^. PAPγ shares greater than 60% identity to the well-characterized PAPα at the amino acid level and is thought to have arisen by gene duplication of the latter^31,32^. However, PAPγ is localized exclusively in the nucleus while PAPα exhibits both nuclear and cytoplasmic localization. The difference in subcellular localization is thought to be due to signals present in the unique C-terminal region (amino acids 507-736) of PAPγ^31–33^. PAPγ carries out both nonspecific and CPSF/AAUAAA-dependent polyadenylation activity. The catalytic efficiency of PAPγ is similar to that of PAPα. PABPN1 acts as a coactivator of both PAPα and PAPγ. Indeed, PABPN1 plays a role in RNA polyadenylation by strongly increasing the processivity of poly(A) polymerases, leading to hyperadenylation of RNA targets with the addition of up to 800 adenosines^26,27,29^. Interestingly, the nuclear exosome has been shown to be involved in PABPN1 and PAP-mediated decay of intronless β-globin and PANΔENE reporters^26^.

TRAMP is a nuclear polyadenylation complex that was initially characterized in *Saccharomyces cerevisiae*^19,35^. It is composed of a non-canonical PAP, TRF4p, together with MTR4p helicase and a zinc knuckle protein, Air2p. TRF4p adds a short polyA tag to the 3’ end of the target RNA, which is required for its degradation by the nuclear exosome^19^. Such a mechanism was suggested for the NEXT complex via PAPD5/ZCCHC7 subunits^22^. However, it is unclear how PAXT substrates become polyadenylated. PAXT substrates harbor a long polyA tail that is bound by PABPN1 to facilitate recruitment of PAXT. In addition, PROMPTs were resistant to degradation by the exosome when cells were treated using cordycepin, an inhibitor of polyadenylation, highlighting the importance of the polyA tail for degradation. However, the specific polyA-polymerase involved has not been described.

*S. pombe* Mtl1-Red1 Core (MTREC) is an 11-subunit complex that is thought to be homologous to PAXT^36^. MTREC consists of several modules that are bridged by Red1, which acts as a scaffold. Red1 has been proposed to be the homolog of human ZFC3H1. MTR4, and ZC3H3 are the homologs of MTL1 and Red5, respectively, while both RBM27 and RBM26 are homologs of Rmn1. Interestingly, MTREC contains a polyA binding protein, Pab2 that is a homolog of PABPN1, and a polyA polymerase, Pla1, for which the canonical polyA-polymerases, PAPα and PAPγ, have been proposed as homologs^37^. To date, no direct link between a polyA polymerase and PAXT complex has been described. However, it was shown that both PABPN1 and canonical polyA-polymerases are involved in the decay of mRNA, such as those with retained introns, and PROMPTs. However, no distinction was made between the canonical polyA polymerases, PAPα and PAPγ^26,27^.

In this study, we sought to address the mechanisms underlying the processing of RNA targets by PAXT. Using proteomics of immunopurified ZFC3H1, we identified the polyA polymerase PAPγ as the sole PAP detected in the PAXT complex. We mapped for the first time the localization of subunits of PAXT on chromatin using ChIP-seq. We found that PAPγ co-localizes with PAXT subunits, ZFC3H1 and RBM26, at the TSS of hundreds of genes. Importantly, ZFC3H1 is required for PAPγ recruitment at these sites. RNA-seq analysis showed that loss of ZFC3H1 or PAPγ was associated with the accumulation of PROMPTs at genes directly targeted by PAXT. We further showed that PAXT, including PAPγ, is implicated in the processing and polyadenylation of PROMPTs. Thus, we demonstrate that PAPγ associates with PAXT and is essential for polyadenylation and subsequent degradation of PROMPTs. This study uncovers a connection between the nuclear polyA polymerase, PAPγ, and the PAXT complex that contributes to the processing of PROMPTs. It further indicates that PAXT substrates such as PROMPTs are likely targeted and possibly degraded at their site of production on chromatin.

## Results

### PAPγ is a component of the PAXT complex

**Fig. 1:**
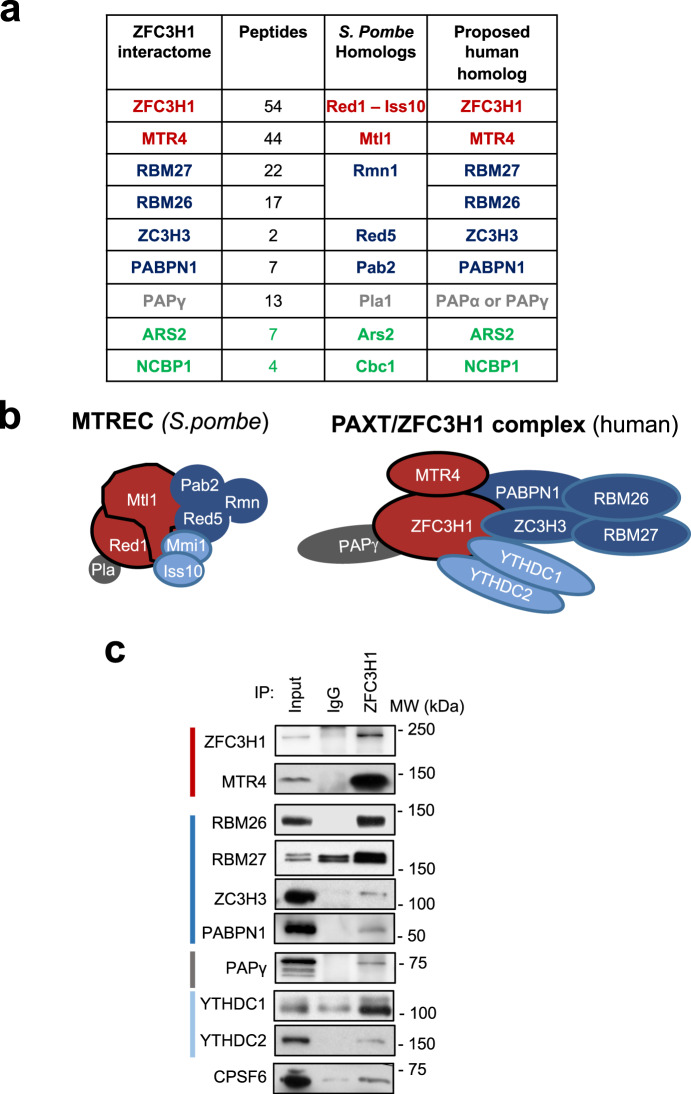
PAPγ is a component of the PAXT complex. **a** Table showing MTREC subunits found in *S. pombe*, the proposed human homologs, the corresponding proteins identified in the ZFC3H1 interactome together the number of unique peptides identified by mass spectrometry. **b** Schematic model depicting the MTREC complex in *S. pombe* (top) and the human PAXT complex based on proteins identified in the ZFC3H1 interactome. **c** Co-immunoprecipitation analysis of ZFC3H1. HeLa nuclear extracts were immunoprecipitated using antibodies against ZFC3H1 or an IgG control. Immunoprecipitates and an aliquot of nuclear extract (input, 5%) were analyzed by SDS-PAGE followed by immunoblot using the antibodies indicated on the figure. Molecular weight markers in kDa are shown at right. Shown is a representative result taken from 3 independent experiments. Source data are provided as a Source Data file.

In order to gain insight into PAXT function, we first created a HEK-293T cell line expressing ZFC3H1 with an N-terminal Flag-HA (FH) tag using Crispr-Cas9 technology to insert the tag at the endogenous locus. FH-ZFC3H1 interacted with a known ZFC3H1 partner, MTR4, as expected (Supplementary Fig. 1A). FH-ZFC3H1 was purified from nuclear extracts by tandem affinity purification. Compared to control cells, a band at the expected size for FH-ZFC3H1 could be distinguished in a silver-stained gel (Supplementary Fig. 1B). Analysis of tandem affinity purified FH-ZFC3H1 by mass spectrometry identified 131 interactants having 2 or more peptides and FC > 2 compared to the control HEK-293T sample. Among the most represented pathways obtained using gene ontology analysis were RNA processing and splicing (Supplementary Fig. 1C). Polyadenylation was also significantly overrepresented, which might be expected given the connection of PAXT with polyadenylation. Among the interactants, ZFC3H1 and exosome subunits such as MTR4, but not ZCCHC8, were detected (Supplementary Table S1), which is consistent with a previous study^23^. Interestingly, all proposed human orthologs of the yeast MTREC complex were strongly represented among ZFC3H1 interactants (Fig. 1a, b). Red1 found in *S. pombe* has been proposed to be the homolog of human ZFC3H1. MTR4, ZC3H3, and PABPN1 are the homologs of Mtl1, Red5, and Pab2, respectively, while both RBM26 and RBM27 are homologs of Rmn1. Interestingly, however, the sole homolog identified for the polyA polymerase Pla1 was PAPγ, a strictly nuclear canonical polyA polymerase. Notably, although PAPα and PAPγ share redundant functions^26,27^, the ZFC3H1 interactome contained only PAPγ (Supplementary Data 1). PAPα was not detected among ZFC3H1 interactants.

In order to confirm interactions, we next performed co-immunoprecipitation analysis of endogenous, unmodified proteins present in HeLa nuclear extracts (Fig. 1c and Supplementary Fig. 2A). As expected, ZFC3H1 was found to interact with subunits of PAXT, such as MTR4, RBM26, and RBM27, as well as PABPN1. Notably, the interaction between ZFC3H1 and PAPγ was confirmed by co-immunoprecipitation. PAPγ was furthermore found to interact with PAXT subunits RBM26, RBM27 and PABPN1 (Supplementary Fig. 2A). RBM26 and RBM27 were also shown to interact with PAXT subunits (Supplementary Fig. 2A). In addition to PAPγ and PABPN1, proteomic analysis identified other ZFC3H1 interactants involved in polyA processing, including CPSF6 and a polyadenosine RNA-binding protein, ZC3H14, also known as MSUT2. Their interaction with ZFC3H1 was confirmed by co-immunoprecipitation (Fig. 1c and Supplementary Fig. 2A). Although Mmi1 and Iss10 homologs, YTHDC1 and YTHDC2, were not identified among the interactants, a previous study identified YTHDC1 as an interactant of ZFC3H1 upon over-expression^23^. Co-IP analysis confirmed that both m6A readers, YTHDC1 and YTHDC2, interacted with endogenous PAXT subunits, including PAPγ (Fig. 1c and Supplementary Fig. 2A). To determine whether PAXT subunits interact in an RNA-dependent manner, we performed co-immunoprecitation analysis in the presence and absence of RNAse. Notably, interaction of PAPγ with ZFC3H1, MTR4 and RBM27 was independent of RNA, whereas interaction between PAPγ and PABPN1 was found to be RNA-dependent. Similar results were obtained for ZFC3H1 (Supplementary Fig. 2B). Taken together, these data identify additional partners of the ZFC3H1 subunit of the PAXT complex. In particular, the proposed orthologs of yeast MTREC subunits were identified and/or confirmed by co-immunoprecipitation as interactants of endogenous ZFC3H1.

### PAXT subunits are co-recruited to chromatin

**Fig. 2:**
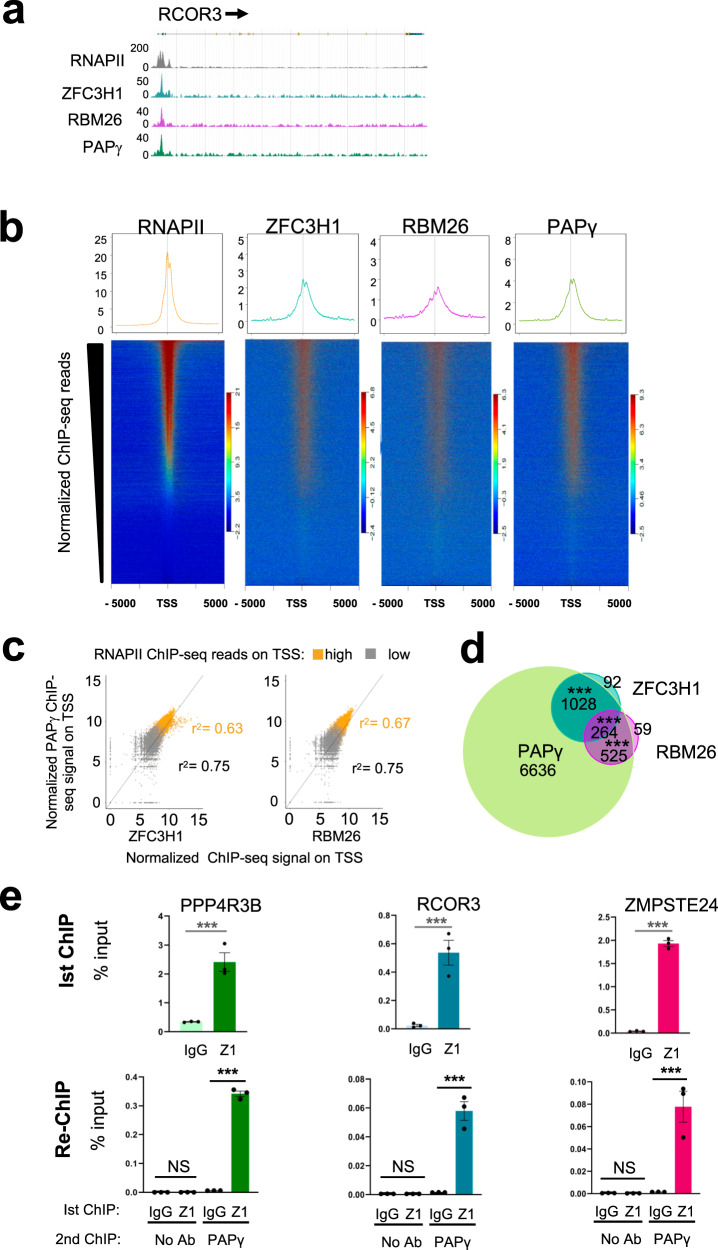
PAXT subunits are co-recruited to chromatin. **a** Browser shots of RNAPII, ZFC3H1, RBM26, and PAPγ ChIP-seq signal over a representative gene in HeLa cells. A schematic representation of the gene is shown above. **b** ChIP-seq heatmaps centered on TSSs ± 5 kb and rank-ordered by normalized RNAPII ChIP-seq signal. Normalized ChIP-seq reads of ZFC3H1, RBM26, or PAPγ were plotted respecting the same ranking. Scaled average density profiles of normalized ChIP-seq reads are shown above. **c** Scatter plots showing the normalized ChIP-seq signal of PAPγ relative to that of ZFC3H1 or RBM26 at the TSS of genes with a high or low occupancy of RNAPII at the TSS, as indicated. **d** Venn diagram showing the overlap of ChIP-seq peaks of ZFC3H1, RBM26, and PAPγ. *P*-values were calculated using Fisher’s exact test (^***^P < 0.001). **e** Re-ChIP analysis performed in HeLa cells showing co-localization of ZFC3H1 and PAPγ at the TSS region of the indicated genes. Eluates from an initial ChIP using ZFC3H1, or a control IgG antibody (1st ChIP) were used for PAPγ ChIP or no antibody as a control (re-ChIP). Results of qPCR after 1st and 2nd ChIPs are shown as % of input for 1st ChIP. Data represent mean ± SEM obtained from 3 independent experiments (***P < 0.001, NS indicates not significant, one-sided independent Student’s *t*-test). Source data are provided as a Source Data file.

RNA processing, including polyadenylation, frequently occurs co-transcriptionally^38^ and the recruitment of RNA-binding proteins, such as splicing factors, can be monitored by chromatin immunoprecipitation (ChIP)^39^. Consistent with this, cellular fractionation analysis revealed that PAXT subunits, MTR4, ZFC3H1, RBM26, RBM27, and PAPγ, partially localized to chromatin in addition to the nucleoplasm (Supplementary Fig. 3A). To better characterize PAXT and its functions, we sought to identify sites of recruitment of some of its key subunits on chromatin. ChIP-seq was performed using antibodies recognizing known PAXT subunits, ZFC3H1 and RBM26, as well as PAPγ, in addition to RNA polymerase II (RNAPII). It should be noted that antibodies against RBM27 did not yield an analyzable signal. ChIP-seq reads of ZFC3H1, RBM26 and PAPγ were detected mostly over gene bodies and transcription start sites (TSS) as well as at enhancers. The distribution was similar to that observed for RNAPII (Supplementary Fig. 3B). At genes, the signal was more intense at TSSs and was characterized by a sharp peak (Fig. 2a and Supplementary Fig. 3C). The specificity of PAPγ ChIP-seq signal was confirmed by RNAi-ChIP-qPCR at the TSS of target genes using the same antibody used in ChIP-seq as well as a second independent antibody recognizing PAPγ (Supplementary Fig. 3D).

We next addressed whether PAXT subunits bind chromatin as a protein complex. The presence of both ZFC3H1 and PAPγ could be detected at TSSs that are associated with PROMPTs, as shown by ChIP-qPCR (Supplementary Fig. 4A). ZFC3H1, RBM26, and PAPγ ChIP-seq signals were then mapped at TSSs that had been ranked according to RNAPII occupancy. The heatmaps showed that all 3 subunits were associated with an overlapping subset of TSSs that could be largely ranked by RNAPII signal intensity (Fig. 2b). Indeed, binding sites of PAPγ were highly correlated with those of ZFC3H1 or RBM26 as well as with RNAPII (Fig. 2c). Regions bound by ZFC3H1, RBM26 or PAPγ were also bound by RNAPII (Supplementary Fig. 4B) although the correlation with RNAPII occupancy was better for RBM26 and PAPγ compared to ZFC3H1, which may be due to detection thresholds of the different antibodies used.

Further analysis confirmed that peaks of PAPγ, RBM26, and ZFC3H1 also significantly overlapped (Fig. 2d). Indeed, almost all peaks of ZFC3H1 (93,3%, *P*-value < 1e-79) and RBM26 (93%, *P*-value < 1e-51) overlapped with peaks of PAPγ. It should be noted however that many peaks of PAPγ did not overlap with those of ZFC3H1 or RBM26, which could be due to lower detection efficiency of these latter factors due to lower efficiencies of the antibodies in immunoprecipitating such complexes, or the association of PAPγ with complexes independent of PAXT.

To further confirm co-recruitment of ZFC3H1 and PAPγ at TSSs, we performed re-ChIP experiments. TSSs associated with ZFC3H1 (1st ChIP) were found to be associated with PAPγ (re-ChIP), demonstrating that both ZFC3H1 and PAPγ are co-recruited at these sites (Fig. 2e). Thus, these results show that PAXT subunits ZFC3H1, RBM26 and PAPγ are recruited to chromatin, at TSSs as well as at enhancers, whose associated RNAs have been shown to be exosome substrates^40,41^. These data furthermore reveal that ZFC3H1, RBM26 and PAPγ frequently co-localize on chromatin, likely as the PAXT complex, at sites of active transcription.

**Fig. 3:**
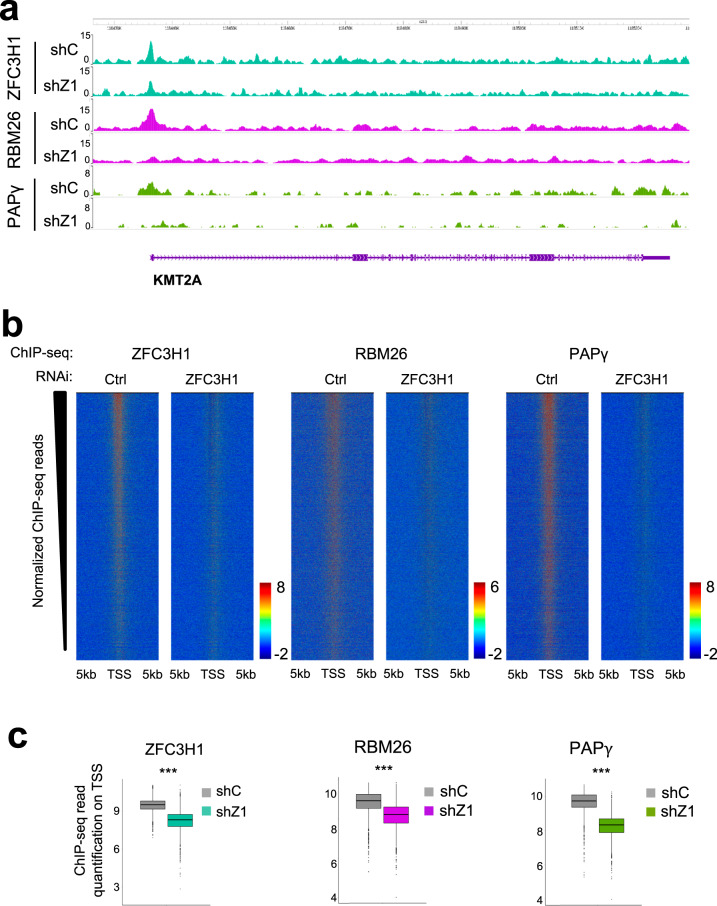
ZFC3H1 is required for recruitment of PAXT to chromatin. **a** Browser shots of ZFC3H1, RBM26, and PAPγ ChIP-seq signal over a representative gene in HeLa cells following loss of ZFC3H1 or a control. A schematic representation of the gene is shown below. **b** Heatmaps centered on TSSs ± 5 kb showing normalized ChIP-seq reads of ZFC3H1, RBM26, or PAPγ in shCon and shZFC3H1 samples and rank-ordered by the change in ZFC3H1 signal between shCon and shZFC3H1 biological replicate samples. Normalized ChIP-seq reads of RBM26 or PAPγ were plotted respecting the same ranking. **c** Box plot quantification of normalized ChIP-seq reads of ZFC3H1, RBM26, or PAPγ in shCon and shZFC3H1 samples, as indicated, at the top decile of the heatmaps shown in b. The box plots display the median, upper, and lower quartiles; the whiskers show a 1.5× interquartile range (***P < 0.001, Wilcoxon test, *n* = 1808).

To further investigate recruitment of PAXT to chromatin, we depleted a major targeting subunit, ZFC3H1, and performed ChIP-seq experiments for PAXT subunits. Immunoblot analysis of extracts confirmed depletion of ZFC3H1 but no significant effect on expression of either RBM26 or PAPγ was detected (Supplementary Fig. 5). ZFC3H1 depletion was accompanied by a significant decrease of ZFC3H1 at chromatin, particularly at TSSs (Fig. 3a, top panel, Fig. 3b, left panel, Fig. 3c, left panel *P*-value < 1e-16, Wilcoxon test). Heatmaps of ChIP-seq signal loss over TSSs showed that loss of RBM26 and PAPγ was largely correlated to loss of ZFC3H1. Notably, loss of ZFC3H1 was accompanied by a significant decrease in signal of both RBM26 and PAPγ at TSSs (Fig. 3a and Fig. 3b, middle and right panels, Fig. 3c, middle and right panels, *P*-values < 1e-16, and <1e-16 respectively, Wilcoxon test). While recruitment of RBM26 and PAPγ at PAXT binding sites appears to be dependent on the presence of ZFC3H1, we cannot exclude that recruitment of these factors at other sites via additional factors, or through indirect mechanisms, may also occur. Taken together, these data suggest that ZFC3H1 is implicated in the recruitment of other components of the PAXT complex at the genome-wide level.

### PAXT modulates the abundance of PROMPTs

**Fig. 4:**
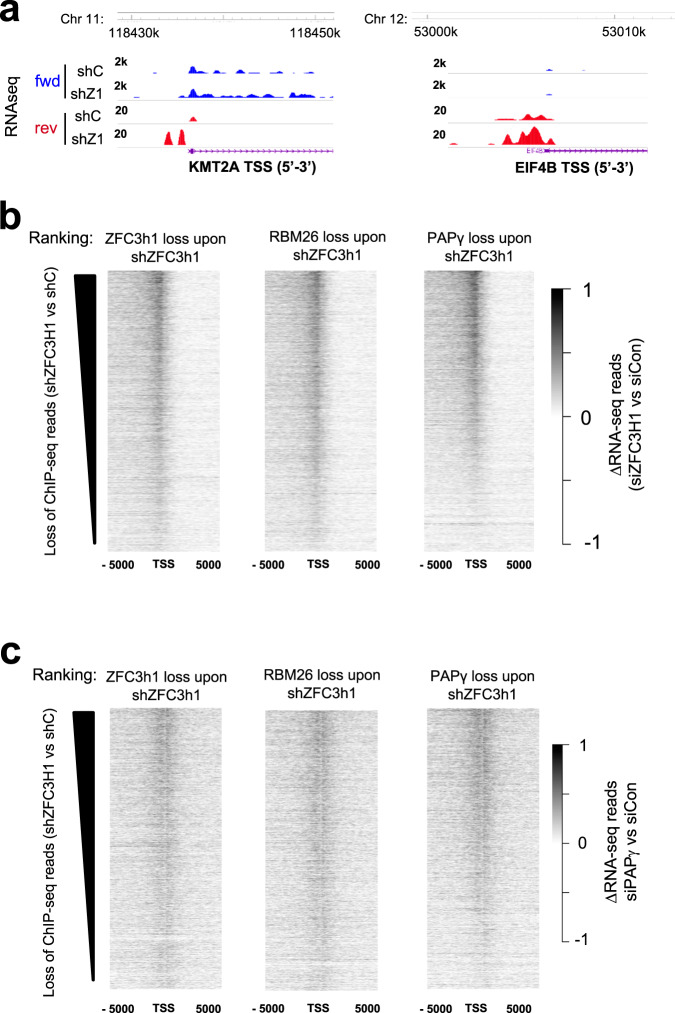
Loss of PAXT subunits leads to the accumulation of PROMPTs genome-wide. **a** Browser shots of RNA-seq reads at the TSS region of representative genes following loss of ZFC3H1 or a control in HeLa cells. A schematic representation of the gene is shown below. **b**, **c** Heatmaps representing the differential of RNA-seq reads following loss of ZFC3H1 (**b**) or PAPγ (**c**) compared to a control knock-down, centered on TSSs ± 5 kb and rank-ordered by change in normalized ChIP-seq reads of ZFC3H1 (left), RBM26 (middle) or PAPγ (right) in HeLa cells.

The association of PAXT at TSSs might indicate that the processing of substrates such as PROMPTs occurs in the vicinity of chromatin. To determine if chromatin-association of PAXT is implicated in the processing of PROMPTs genome-wide, we performed de novo analysis of RNA-seq data in ZFC3H1 depletion condition^25^. TSSs were oriented 5’−3’ and ranked by the loss of ChIP-seq signal of ZFC3H1, RBM26, or PAPγ, following depletion of ZFC3H1. Change in RNA-seq reads were calculated and mapped at the same sites. Strikingly, the highest accumulation of PROMPTs occurred at TSSs showing the greatest loss of PAXT upon depletion of ZFC3H1 (Fig. 4a, b). To determine if these findings extended to PAPγ, RNA-seq was performed in PAPγ-depleted HeLa cells (Supplementary Fig. 6A) and a similar analysis was carried out. As observed for ZFC3H1, loss of PAPγ also led to the accumulation of TSS-associated RNAs, which could be ranked by the loss of ZFC3H1, RBM26, or PAPγ at the sites following depletion of ZFC3H1 (Fig. 4c). Interestingly, loss of PAPγγ did not have a major impact on the abundance of mRNAs (Supplementary Fig. 6B). Only 279 mRNAs were down-regulated and 166 were up-regulated following loss of PAPγ. This finding is consistent with previous studies showing that loss of PAPγ alone did not have a significant effect on RNA abundance^26,27^. Taken together, these results strengthen the importance of PAPγ and other PAXT subunits in the regulation of PROMPTs. They furthermore show that nuclear exosome-associated factors can be detected at their site of activity, suggesting that targeting and possibly degradation occur at the site of transcription.

**Fig. 5:**
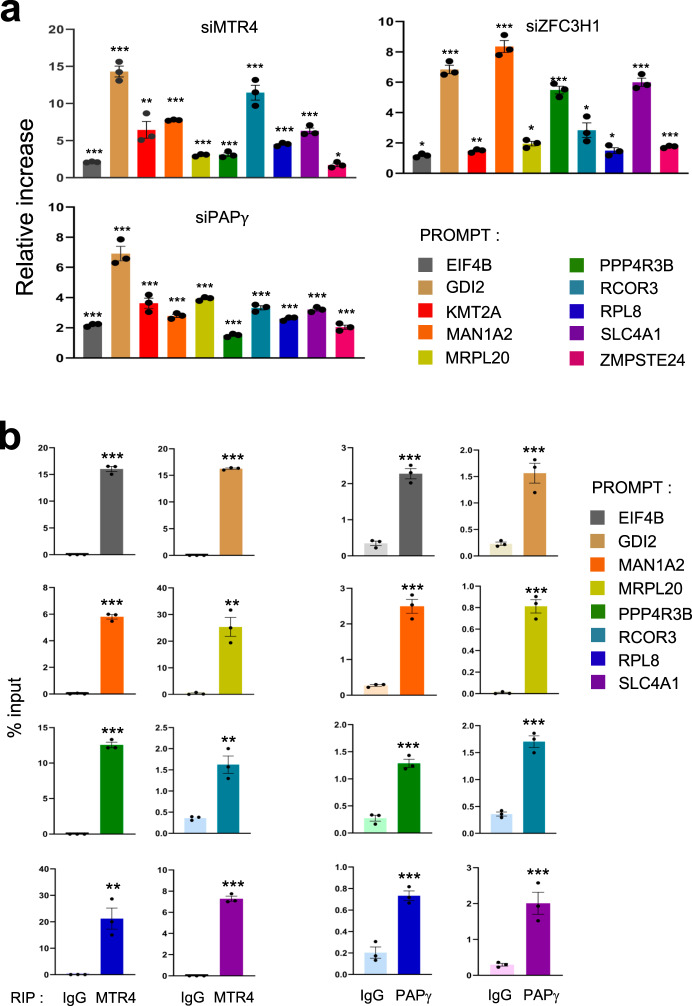
ZFC3H1, MTR4, and PAPγ modulate the abundance of PROMPTs. **a** Total RNA extracts of HeLa cells transfected with siRNAs directed against MTR4, ZFC3H1, PAPγ or a control were analyzed by RT-q-PCR using the oligonucleotide pairs indicated. The values were normalized to those for the control transfection, which was attributed to a value of 1. Data represent mean ± SEM obtained from 3 independent experiments (***P < 0.001, ^**^P < 0.01, ^*^P < 0.05, NS indicates not significant, one-sided independent Student’s *t*-test). **b** Total RNA extracts of HeLa cells were analyzed by RIP using antibody to MTR4, PAPγ, or control IgG, as indicated. Immunoprecipitates and an aliquot of extract were analyzed by RT-qPCR using oligonucleotide pairs indicated and the values were expressed relative to the input sample (%). Data represent mean ± SEM obtained from 3 independent experiments (^***^P < 0.001^**^P < 0.01, ^*^P < 0.05, one-sided independent Student’s *t*-test). Source data are provided as a Source Data file.

To further characterize the function of PAPγ and PAXT in the stabilization of PROMPTs, we performed quantitative reverse transcription PCR (RT-qPCR) for PROMPTs associated with TSSs bound by PAXT. As expected, KD of MTR4 as well as that of ZFC3H1 resulted in significant up-regulation of PROMPTs (Fig. 5a and Supplementary Fig. 7A). Loss of PAPγ also increased PROMPTs levels, similar to depletion of PAXT subunits MTR4 and ZFC3H1. Similar results were obtained using a second independent siRNA targeting either MTR4, ZFC3H1, PAPγ, or a non-targeting control (Supplementary Fig. 7B, C).

To determine whether the PAXT-dependent PROMPTs identified by genome-wide analyses are directly targeted by PAXT complex, we performed RNA immunoprecipitation (RIP) analysis. As shown in Fig. 5b (left panels), PROMPTs were significantly detected in immunoprecipitates of MTR4. Notably, ZFC3H1 co-immunoprecipitated with MTR4, as expected (Supplementary Fig. 7D). Similar results were obtained using anti-PAPγ antibody (Fig. 5b, right panels and Supplementary Fig. 7E). These results demonstrate that PAXT subunits, PAPγ and ZFC3H1 together with MTR4, associate with PROMPTs, which likely occurs at the site of transcription, to modulate their abundance. Taken altogether, our data thus support a mechanism for the direct targeting and degradation of PROMPTs by PAXT at sites of ncRNA production on chromatin.

### PAPγ is implicated in the polyadenylation of PROMPTs

Substrates of the PAXT complex are thought to be polyadenylated, as suggested by the association of PABPN1 with PAXT^23^. However, the identity of the polyA polymerase is not known. Since our data show that PAPγ is associated with the PAXT complex and modulates the abundance of PROMPTs, we sought to determine whether PAPγ might polyadenylate PAXT-dependent PROMPTs.

**Fig. 6:**
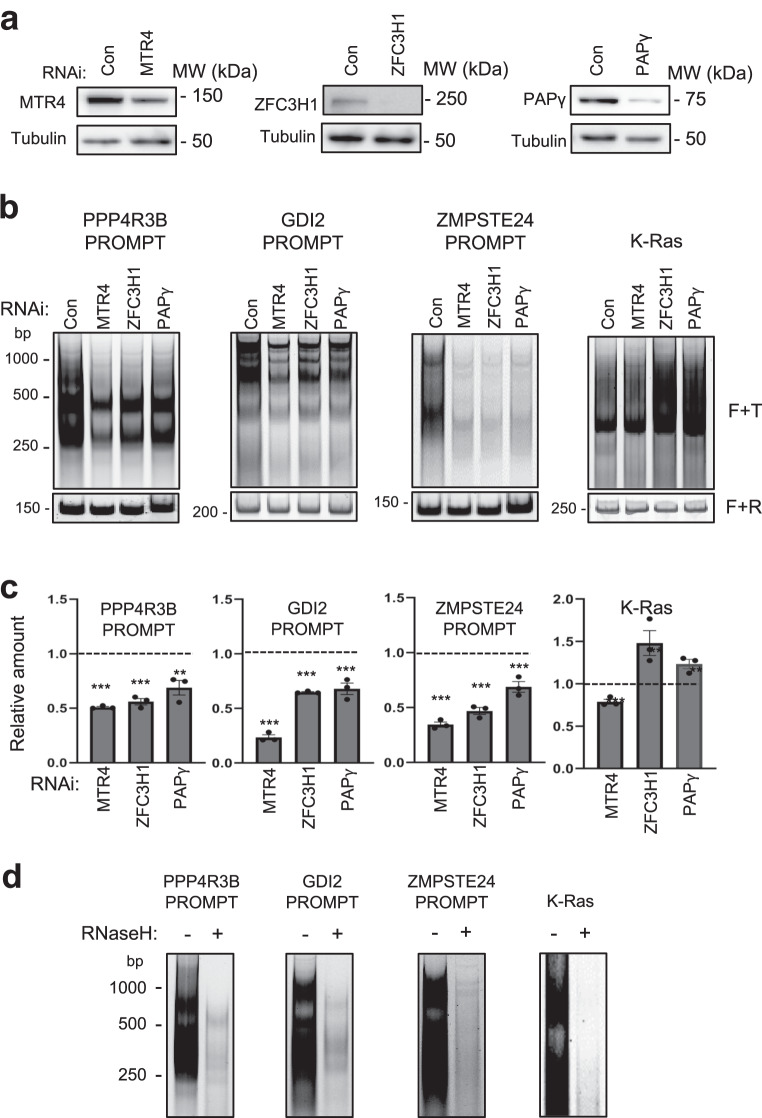
PAPγ is implicated in the polyadenylation of PROMPTs. **a** Immunoblot analysis of HeLa cell extracts following transfection with siRNAs directed against MTR4, ZFC3H1, PAPγ, or a control (con), as indicated on the figure. Samples were analyzed by SDS-PAGE followed by immunoblot using the antibodies indicated on the figure. Molecular weight markers in kDa are shown at right.Shown is a representative result taken from 3 independent experiments. **b** Representative polyacrylamide gels showing electrophoretic mobility of PCR products using extracts of HeLa cells transfected with siRNAs targeting MTR4, ZFC3H1, PAPγ, or a non-targeting control (con). PCRs were performed using a forward primer specific for the gene indicated above the gel together with oligo (dT) (F + T; top panel) or with a specific reverse primer (F + R; bottom panel). Size markers in bp are shown at left. *N* = 3. **c** Quantification of F + T signal of gels, as shown in **b**. Values were expressed relative to the control sample, which was attributed a value of 1. Data represent mean ± SEM obtained from 3 independent experiments (^***^P < 0.001^**^P < 0.01, one-sided independent Student’s *t*-test). **d** Polyacrylamide gels showing electrophoretic mobility of PCR products performed using a specific forward primer together with oligo (dT) (F + T) from extracts of HeLa cells. Samples were treated with RNase H or mock-treated, as indicated on the figure. Shown is a representative result taken from 3 independent experiments. Source data are provided as a Source Data file.

To determine whether PAPγ can polyadenylate PROMPT ncRNAs, we measured polyadenylation by poly(A) test (PAT) assay. The PAT assay detects the polyA tail of a specific RNA target by PCR using an oligodT primer together with a primer containing a sequence near the PAS signal of the target RNA. Polyadenylated RNAs generate PCR products with heterogeneous electrophoretic mobility, appearing as a smear. Extracts of HeLa cells transfected with siRNAs targeting either MTR4, ZFC3H1, PAPγ, or a non-targeting control were used in PAT analysis (Fig. 6a). RNAs of several PROMPTs, as well as a control RNA, K-Ras, were first normalized among the different knock-down conditions using PCR primers detecting an internal product (Fig. 6b, bottom panel, F + R). The polyA tail of each PROMPT was then detected using an internal primer together with an oligo (dT) primer (Fig. 6b, top panel, F + T). Knock-down of MTR4, ZFC3H1, or PAPγ significantly reduced the intensity of the polyA tail signal of PROMPTs. In contrast, the intensity of the polyA tail signal of K-Ras mRNA was slightly reduced by depletion of MTR4, and slightly increased by loss of either ZFC3H1 or PAPγ (Fig. 6b, top panel, F + T, Fig. 6c). The polyA tail signal was largely abolished by treatment of the reaction products with RNaseH in the presence of oligo (dT), confirming the specificity of the reaction for polyA polymers (Fig. 6d). Altogether, our data show that PAPγ, which is associated with PAXT complex, contributes to the polyadenylation of PROMPTs.

## Discussion

The nuclear RNA exosome is the major RNA degradation machinery in the nucleus. However, it must be recruited to its target RNAs by one or more adapter complexes. One such complex, PAXT, recruits nuclear exosome to polyadenylated RNAs, via the PABPN1 subunit. Here, we addressed the mechanisms underlying processing of PROMPTs by PAXT. Using proteomics of purified ZFC3H1, we identified PAPγ, which is a predominantly nuclear polyA polymerase, as the sole PAP detected in the PAXT complex. The localization of subunits of PAXT on chromatin was mapped using ChIP-seq. We found that PAPγ co-localizes with PAXT subunits, ZFC3H1 and RBM26, at the TSS of several hundred genes. Importantly, ZFC3H1 is required for PAPγ recruitment at these sites. Finally, we showed that PAPγ was implicated in the processing of PROMPTs and was required for their efficient polyadenylation. Collectively, these data show that a specific PAP, PAPγ, associates with PAXT and is essential for polyadenylation and subsequent degradation of PROMPTs. These findings uncover a connection between the nuclear polyA-polymerase PAPγ and the PAXT complex that contributes to the processing of PROMPTs. It further indicates that certain PAXT RNA substrates, such as PROMPTs, are targeted and likely degraded at their site of production on chromatin.

Polyadenylation is a major RNA processing step occurring on nascent transcripts that determines the fate of cellular mRNAs (see ref. ^42^ for review). In mammals, most mRNAs are polyadenylated, which consists of the addition of ∼250 non-coded adenosines. Polyadenylation confers stability to the mRNA and is required for efficient translation. On the contrary, in prokaryotes, the addition of a polyA tail marks mRNAs for degradation^43–46^. The addition of a short polyA tail (15–40 adenosines) on bacterial transcripts provides a platform for the 3′-exonuclease polynucleotide phosphorylase (PNPase) to initiate 3′–5′ exonucleolytic degradation^47^. Bacterial polyadenylation therefore primarily regulates turnover and quality control of specific cellular transcripts^48^. Polyadenylation of nuclear transcripts could therefore be considered to serve a similar function in mammals^19^. The polyA tail added by the TRAMP complex provides a landing pad for nuclear exosomes to facilitate 3’−5’ RNA degradation or trimming^19,49^. However, the TRAMP complex in humans is localized in the nucleolus^22^ and so it was unclear how nucleoplasmic exosome substrates become polyadenylated. PAPγ has been shown to polyadenylate many snRNAs^26,27^, which are also targeted by PAXT^23,50^. Our findings indicate that PAPγ can also polyadenylate PROMPTs that are targeted by PAXT for degradation by nuclear exosome. This finding supports the recent report showing that Pla1 subunit of MTREC hyperadenylates PROMPTs in *S. pombe*^51^. Interestingly, we observed that loss of the canonical polyA polymerase, PAPα, also affected the abundance of PROMPTs, and showed a cumulative effect in combination with the loss of PAPγ. However, PAPα did not interact with PAXT in either the presence or absence of PAPγ, suggesting that its effect on PROMPTs does not occur via PAXT. Since concomitant loss of both PAPs has been shown to affect the stability of several RNA species^26,27^, it will be interesting to determine whether PAPα and PAPγ might co-operate in the hyperadenylation of PROMPTs through PAXT-dependent and -independent mechanisms.

ZFC3H1 can be found in foci in the nucleus together with polyadenylated RNAs^24^, suggesting that degradation of the substrate RNAs may occur in the nucleoplasm. Using ChIP-seq, we found that several subunits of PAXT, such as ZFC3H1, RBM27, and PAPγ, were associated with chromatin at sites of transcription of PROMPTs. This suggests that PROMPTs are likely targeted by PAXT directly at the site of transcription. Whether degradation also occurs on site, or in foci in the nucleoplasm remains unclear.

Polyadenylation and degradation of unstable nuclear RNAs, such as PROMPTs, is important to prevent their deleterious accumulation. Indeed, Manley and colleagues previously showed that polyadenylated PROMPTs and prematurely terminated transcripts that were stabilized by depletion of either MTR4 or ZFC3H1, but not NEXT subunits, accumulated in the nucleus and were also exported to the cytoplasm where they became associated with polysomes^4^. MTR4- or ZFC3H1-depleted cells displayed significant inhibition of translation of mRNAs, likely due to competition with the exported PROMPTs. Thus, the MTR4 and ZFC3H1-containg complex was therefore termed ‘polysome protector complex’ since it assures the proper polyadenylation and subsequent degradation of PROMPTs and prematurely terminated transcripts in the nucleus, thereby preventing their export to the cytoplasm and occupancy of ribosomes^4^. Our data show that loss of PAXT leads to a 2 to 3-fold reduction of polyA tails, as measured by PAT assay, when PROMPTs have been normalized for abundance. However, depletion of PAXT leads to a significant increase in the abundance of PROMPTs in cells, as measured by RT-qPCR. Therefore, it is likely that, although less efficiently polyadenylated, the significant increase in PROMPT abundance is likely sufficient to lead to cytoplasmic export and deregulation of translation as observed previously^4^.

It was previously shown that asymmetric sequence determinants flanking TSSs control promoter directionality by regulating cleavage and polyadenylation of promoter-proximal transcripts^52^. PROMPT/uaRNAs are enriched for PAS signals while being depleted for U1 sites. It was shown that PROMPTs/uaRNAs are cleaved and polyadenylated at poly (A) sites close to the TSS, with a peak of cleavage sites about 700 bp upstream of the TSS, that were associated with the canonical 3’ end processing machinery. Interestingly, a previous study also showed that polyadenylated PROMPTs detected following depletion of MTR4 or ZFC3H1 contain canonical PAS at the 3’ end^4^. Moreover, we confirmed an interaction between ZFC3H1 and CPSF6 subunit of the 3’-end processing machinery. PAPγ is a canonical poly(A) polymerase. It contains a U1 interaction region in its C-terminus and can be inhibited by U1^31^. PAPγ is highly active in both AAUAAA- and CPSF-dependent polyadenylation, as shown using in vitro polyadenylation assays^31,33^. Therefore, the high density of PAS and the relative paucity of U1 sites in PROMPTs would favor polyadenylation of cleaved PROMPTs by PAPγ. Taken together, these findings suggest that the enrichment of PAS in PROMPTs/uaRNAs facilitates transcript cleavage while the association of PAXT with PAPγ subunit of PAXT, which associates with PROMPT RNAs, carries out polyadenylation. PAXT may then target the polyadenylated transcripts to the nuclear exosome for degradation.

Altogether, our data shed light on the processing of PROMPT ncRNAs. PROMPTs were recently shown to be implicated in transcriptional activation at estrogen-responsive genes by regulating transcriptional pause release^5^. Thus, the identification of factors and mechanisms that control the abundance of PROMPT ncRNAs is important to better understand the mechanisms controlling transcription of the coding genome.

## Methods

### Cell culture and reagents

HeLa cells (ATCC, CCL-2), that are commonly used in molecular biology, were grown in Dulbecco’s modified Eagle’s minimal essential medium (DMEM) (Sigma-Aldrich, D6429), supplemented with 10% fetal calf serum (FCS; Eurobio Scientific, CVFSVF00-01) and containing 1% penicillin-streptomycin (Sigma-Aldrich, P4333). HEK-293T (ATCC, CRL11268) were grown in Hepes-modified DMEM (Sigma-Aldrich, D6171), supplemented with 10% FCS (Eurobio Scientific, CVFSVF00-01) and containing 1% penicillin-streptomycin (Sigma-Aldrich, P4333). All cells were grown in a humidified incubator at 37 °C with 5% CO2. Cell lines were not authenticated.

### Antibodies

Antibodies used in this study are shown in Table S1.

### CRISPR-Cas9 Mediated Editing of endogenous ZFC3H1 gene

An sgRNA targeting the ZFC3H1 gene around the ATG translation start site was cloned in pSpCas9 (BB)−2A-GFP plasmid (Addgene #48138). The plasmid was then transfected into HEK-293T cells along with a single-stranded oligodeoxynucleotide (ssODN) (Table S2) harboring the Flag-HA sequence flanked by homology sequences to ZFC3H1 around the cleavage site. Single cells were isolated and amplified. HEK-293T clones expressing Flag-HA ZFC3H1 were identified by PCR and confirmed by sequencing as well as Western blot using anti-HA and anti-Flag antibodies.

### RNAi

Production of short hairpin RNA (shRNA)-expressing lentiviral particles was performed using plasmids expressing shRNAs targeting ZFC3H1 (Sigma-Aldrich MISSION shRNA, TRCN0000130498) or a non-targeting control (Addgene, plasmid 1864), according to the manufacturer’s instructions. For knockdown experiments, HeLa cells were either transduced with lentiviral particles and harvested 5 days later, or transfected with siRNAs shown in Table S3 using Interferin (PolyPlus) according to the manufacturer’s instructions, and harvested 72 h later.

### ZFC3H1 protein complex purification

ZFC3H1 complex was purified from Dignam high salt nuclear extracts (10.17504/protocols.io.kh2ct8e) from HEK-293T cells stably expressing Flag-HA-ZFC3H1 by two-step affinity chromatography (10.17504/protocols.io.kgrctv6). Sequential Flag and HA immunoprecipitations were performed on equal amounts of proteins. Silver staining was performed according to the manufacturer’s instructions (SilverQuest, Invitrogen). Elutions were precipitated using ProteoExtract Protein precipitation kit (Millipore) according to the manufacturer’s instructions.

Mass spectrometry was performed at the Taplin Facility, Harvard University, Boston, MA. Briefly, precipitates were resuspended in 50 μL ammonium bicarbonate solution (50 mM) with 10% acetonitrile by gentle vortexing. Ten microliters of modified sequencing-grade trypsin (20 ng/μL; Promega, Madison, WI) was added and samples were then placed in a 37 °C room overnight. Samples were acidified with 5 μL of formic acid solution (20%) and then desalted by STAGE tip^53^.

On the day of analysis, the samples were reconstituted in 5–10 µL of HPLC solvent A (2.5% acetonitrile, 0.1% formic acid). A nano-scale reverse-phase HPLC capillary column was created by packing 2.6 µm C18 spherical silica beads into a fused silica capillary (100 µm inner diameter *x* ~ 30 cm length) with a flame-drawn tip^54^. After equilibrating the column, each sample was loaded onto the column using a Famos autosampler (LC Packings, San Francisco CA). A gradient was formed and peptides were eluted with increasing concentrations of solvent B (97.5% acetonitrile, 0.1% formic acid).

As peptides eluted, they were subjected to electrospray ionization and then entered into an LTQ Orbitrap Velos Pro ion-trap mass spectrometer (Thermo Fisher Scientific, Waltham, MA). Peptides were detected, isolated, and fragmented to produce a tandem mass spectrum of specific fragment ions for each peptide. Peptide sequences were determined by matching protein databases with the acquired fragmentation pattern by the software program, Sequest (Thermo Fisher Scientific, Waltham, MA)^55^. All databases include a reversed version of all the sequences and the data was filtered to between a one and two percent peptide false discovery rate.

### Co-immunoprecipitation analysis

Co-immunoprecipitation was performed using nuclear extracts of HeLa cells. Cells were lysed in ice-cold hypotonic buffer [20 mM tris (pH 7.6), 10 mM KCl, and 1.5 mM MgCl_2_] supplemented with EDTA-free complete protease inhibitor mixture (Roche) for 15 min on ice. NP-40 was added at 0.5% final, and extracts were centrifuged 1 min at 14,000 x *g* at 4 °C. The pellet (nuclei) was resuspended in nuclease buffer [20 mM tris (pH 7.6), 150 mM NaCl, 1.5 mM MgCl2, 2.5 mM CaCl2, and 0.5 μL of 100 mM phenylmethylsulfonyl fluoride] and incubated with micrococcal nuclease (2 × 10^3^ U/mL; New England Biolabs) for 2 h at 4 °C. Lysates were cleared by centrifugation at 14,000 x *g* at 4 °C for 10 min and diluted in immunoprecipitation buffer [50 mM tris (pH 7.6), 150 mM NaCl, and 1% NP-40] supplemented with protease inhibitors. Protein concentration was determined using the Bradford reagent (Bio-Rad). Immunoprecipitations were performed using 400 μg of protein extracts with the indicated antibodies (2 μg) and rotated overnight at 4 °C. Protein A Dynabeads were washed three times in immunoprecipitation buffer, added to protein extracts/antibody solution, and incubated for 2 h at 4 °C. Immunoprecipitates were washed extensively with the immunoprecipitation buffer. Where indicated, samples were incubated with RNAse A/T cocktail (Invitrogen AM2286; 1.2 μL/mL of immunoprecipitation buffer) for 30 min at RT on a rotating wheel, followed by 5 washes with the immunoprecipitation buffer. Samples were resuspended in protein sample loading buffer, boiled for 5 min, and analyzed by Western blotting using the antibodies shown in Table S1.

### Cell fractionation analysis

HeLa cells were seeded in 150 mm culture dishes the day prior to protein extraction. Cytoplasmic proteins were extracted using a mild lysis buffer (10 mM Hepes pH 7.9, 10 mM KCl, 0.1 mM EDTA pH 8.0, 2 mM MgCl2, 1 mM DTT, EDTA-free protease and phosphatase inhibitor). The cell pellet was incubated for 10 min on ice, adding 0.07% NP-40 and incubating for an additional 10 min on ice. After centrifugation (2000 x *g*, 5 min, 4 °C), cytoplasmic fraction was collected. The pellet was washed with ice-cold PBS supplemented with protease and phosphatase inhibitors. After centrifugation (2000 x *g*, 5 min 4 °C), the supernatant was discarded leaving the packed nuclear volume (PNV). For extraction of nuclear soluble proteins, nuclei were resuspended drop-wise in 1x PNV of hypotonic buffer (20 mM Hepes pH 7.9, 20 mM NaCl, 1 mM EDTA pH 8.0, 1.5 mM MgCl_2_, 10% glycerol, 1 mM DTT, EDTA-free protease and phosphatase inhibitor), followed by the addition of 1x PNV of high salt buffer (20 mM Hepes pH 7.9, 800 mM NaCl, 1 mM EDTA pH 8.0, 1.5 mM MgCl_2_, 10% glycerol, 1 mM DTT, EDTA-free protease and phosphatase inhibitor). Tubes with the samples were rotated on a wheel for 20 min at 4 °C, followed by centrifugation (20 min 16,000 x *g*, 4 °C). Supernatant containing the nuclear soluble fraction was collected. The pellet was measured and chromatin-bound proteins were extracted by adding 2 volumes of mild salt buffer (20 mM Hepes pH 7.9, 150 mM NaCl, 1 mM EDTA pH 8.0, 1.5 mM MgCl_2_, 10% glycerol, 1 mM DTT, EDTA-free protease and phosphatase inhibitor) together with 250 U Benzonase (Sigma-Aldrich) per mL. Samples were incubated for 15 min at 37 °C on a rotating wheel. After centrifugation (15 min, 16,000 x *g*, 4 °C), the chromatin-bound fraction was collected. Proteins were analyzed by Western blotting using the antibodies shown in Table S1.

### Quantitative RT-PCR

Total RNA was extracted from HeLa cells using TRIzol (ThermoFisher Scientific) according to the manufacturer’s instructions. Extracts were treated with DNase I (Promega) and reverse transcribed using SuperScript III First-Strand Synthesis System (ThermoFisher Scientific). RT products were amplified by real-time PCR (LightCycler™ 480, Roche) using SYBR Green I Master mix (Roche) with the indicated oligonucleotides. Q-PCR cycling conditions are available on request. Sequences of qPCR primers used in this study are shown in Table S2.

### RNA Immunoprecipitation

RIP was performed as previously described^56^. Briefly, HeLa cells were seeded in 100 mm culture dishes and incubated overnight at 37 °C. Cells were lysed for 10 min in RIP buffer (20 mM HEPES, pH 7.5, 150 mM NaCl, 2.5 mM MgCl_2_•6H_2_O, 250 mM sucrose, 0.05% (v/v) NP-40 and 0.5% (v/v) Triton X-100) containing 20 U mL^−1^ of RNasin (Promega), 1 mM DTT, 0.1 mM PMSF and EDTA-free protease and phosphatase inhibitor. After centrifugation (16 000 x g 10 min at 4 °C), lysates were incubated for overnight at 4 °C with 2 μg of antibodies recognizing MTR4 or IgG control and then incubated for 1 h at 4 °C with Dynabeads™ Protein A (ThermoFisher Scientific). After incubation, beads were washed five times with RIP buffer for 5 min at 4 °C and RNA was extracted using TRIzol (Thermo Fisher Scientific) according to the manufacturer’s instructions. RNA was treated with DNAse I (Promega) and RT was performed using SuperScript™ III Reverse Transcriptase (ThermoFisher Scientific) according to the manufacturer’s instructions. cDNAs were used to perform qPCRs using LightCycler™ 480 SYBR Green I Master mix (Roche), according to the manufacturer’s instructions, using the primers shown in Supplementary Table S2.

### RNA-seq

For RNA-seq, total RNA was extracted from HeLa cells using TRIzol (Thermo Fisher Scientific) according to the manufacturer’s instructions. RNA-seq (paired-end, 125 bp) was carried out by Novogene in triplicates.

### ChIP, library preparation, and sequencing

Chromatin immunoprecipitation followed by high throughput sequencing (ChIP-seq)^57^ was performed as described previously^58^ from HeLa cells using the ChIP-IT High Sensitivity Kit from Active Motif (reference 53040) according to the manufacturer’s instructions. Briefly, sonication was performed using the Qsonica Q700 Sonicator with microtip of 1/8 inches (reference 4418) at 11% amplitude and 13 min of processing time (30-s “ON” and 30-s “OFF”). Each ChIP used 30 μg of chromatin together with 4 μg of antibody detecting ZFC3H1, RBM26, PAPγ or RNAPII (Supplementary Table S1). ChIP-seq libraries were constructed using the Next Gen DNA Library Kit (Active Motif, 53216 and 53264). Library quality was assessed using Agilent 2100 Bioanalyzer and Agilent High Sensitivity DNA assay. Libraries were sequenced with 2^nd^ generation sequencing chemistry on a Nextseq500 (Illumina) at the GENOM’IC facility, Institut Cochin, Paris.

### ChIP and Re-ChIP–qPCR

ZFC3H1 and PAPγ ChIP were performed as previously^59^ using the iDeal ChIP-qPCR Kit (Diagenode, catalog no. C01010180) following the manufacturer’s instructions. Briefly, HeLa cells were sonicated using the Bioruptor Pico (Diagenode, catalog no. B01060001) for 8 cycles of 30-s ON and 30-s OFF at high-power setting. ChIP was performed using the ChIP-IT Express Enzymatic Kit (Active Motif, catalog no. 53009), following the manufacturer’s instructions. Chromatin was digested for 7 min, and 50 μg of chromatin and 3 μg of antibody were used.

Re-ChIP was performed as described previously^58^ using the Re-ChIP-IT Kit (Active Motif, catalog no. 53016) according to the manufacturer’s instruction. Briefly, Chromatin for re-ChIP was prepared using the ChIP-IT High Sensitivity Kit (Active Motif, catalog no. 53040), as described above. For each Re-ChIP, 50 μg of chromatin and 3 μg of antibody were used. Antibodies and sequences of primers used for real-time qPCR analysis are shown in Tables S1 and S2.

### PAT assay

PAT assays were performed as previously described^60,61^. Briefly, total RNA was extracted from cells using TRIzol (ThermoFisher Scientific) according to the manufacturer’s instructions. RNA was treated with RQ1 DNase (Promega) in the presence of RNasin (Promega), then reverse transcribed using SuperScript™ III (Invitrogen™) using either oligo (dT)-anchor (5^’^-GCGAGCTCCGCGGCCGCGTTTTTTTTTTTT-3^’^) or random hexamer primer. For normalization, cDNAs synthesized using random hexamer priming were amplified by quantitative PCR using the Forward and Reverse oligonucleotide pairs indicated in Table S2. Abundance of PCR products was calculated relative to the siCon sample, which was attributed a value of 1. Quantitative PCR reactions, using normalized amounts of cDNA generated using oligo (dT)-anchor priming, were then performed using the forward primer indicated in Table S2 together with oligo(dT)-anchor (50 μM). PCR products were run on 5% non-denaturing polyacrylamide TAE gels, stained with SYBR® Gold and visualized with ChemiDoc MP High-end imaging system.

Where indicated, samples were treated with RNase H. Briefly, DNase-treated RNA was incubated for 15 min at 70 °C with 2 μL of Oligo(dT)20 in a thermocycler and the mixture was slowly cooled down to room temperature. Samples were then incubated for 1 h at 37 °C with 4U RNase H (Invitrogen™) or mock-treated as a control prior to reverse transcription using oligo (dT) primer and qPCR amplification as described above.

### Bioinformatic analyses

For analysis of ChIP-seq data, sequencing reads were first filtered, using fastq_illumina_filter, and quality control of filtered reads was performed using FastQC. Filtered reads were then aligned onto the HG38 genome^62^ using the Burrows-Wheeler Aligner^63^ with default parameters. The sorted BAM files generated by SAMtools^64^ keeping only reads with a mapping quality at least 30 were then normalized by deepTools^65^ bamCoverage function, with a bin size of 10 bp. RPGC normalization was applied, with an effective genome size of 2,913,022,398 bp according to DeepTools user manual instructions. Peak calling was performed using NormR’s enrichR function, searching for enrichment of each BAM file of ChIP-seq reads against the input BAM file using a false discovery rate threshold of 1e-4. Genomic Ranges^66^ was then used to determine overlap between the peak range and genomic features of interest, such as genes with a TSS and TES from GRCh38. Profile matrices were extracted from the normalized data files using DeepTools computeMatrix using a bin size of 10 bp. Profiles were generated +/−5 kbp of TSSs and quantification of normalized reads was performed on +/−500 bp surrounding TSSs. Genomic elements and protein coding genes were obtained from Ensembl (http://www.ensembl.org/Homo_sapiens/Info/Index). Average profiles around genomic TSS were generated using SeqPlots^67^. Heatmaps were generated based on the indicated features with genomation (DOI: 10.18129/B9.bioc.genomation) using the gridHeat function, as performed on profile matrices generated by DeepTools. Proportional Venn diagrams were plotted using ‘Vennerable’ R package (https://github.com/js229/Vennerable).

For expression analyses, RNA-seq data were obtained from GSE131255^25^ or the present study. Forward and reverse RNA-seq reads were filtered using Ensembl reference coding genes to extract only antisense transcripts. Heatmaps were generated with genomation, as for ChIP-seq.

### Statistical analysis

Data presented as histograms are shown as means ± SEM. Comparison between the two groups was analyzed by one-tailed Student’s *t*-test, and asterisks represented significance defined as *P < 0.05, **P < 0.01, or ***P < 0.001. Enrichments in Venn diagrams were performed using Fisher’s exact test. Comparison of ChIP-seq signal in box plots was performed using the Wilcoxon pair-wise test.

### Reporting summary

Further information on research design is available in the Nature Portfolio Reporting Summary linked to this article.

### Supplementary information

Supplementary Information

Description of Additional Supplementary Files

Supplementary Data 1

Reporting Summary

## Data Availability

The data supporting the findings of this study are available from the corresponding authors upon request. ChIP-seq data generated in this study have been deposited at GEO under accession code GSE189157. RNA-seq data were obtained from GSE131255^25^ or the present study (GSE189157). Mass spectrometry data have been deposited at Massive under accession code MSV000089482. Source data are provided as a Source Data file.
