# Supplementary material for: PAPγ associates with PAXT nuclear exosome to control the abundance of PROMPT ncRNAs
Source: Nat Commun. 2023 Oct 24;14:6745. doi: 10.1038/s41467-023-42620-9 (PMC10598014; doi:10.1038/s41467-023-42620-9)
Supplement: Supplementary file 1 — Supplementary Information [file 41467_2023_42620_MOESM1_ESM.pdf]

Supplementary Information for

PAPγ associates with PAXT nuclear exosome to control the abundance of PROMPT ncRNAs

Xavier Contreras, David Depierre, Charbel Akkawi, Marina Srbic, Marion Helsmoortel, Maguelone Nogaret, Matthieu LeHars, Kader Salifou, Alexandre Heurteau, Olivier Cuvier and Rosemary Kiernan*

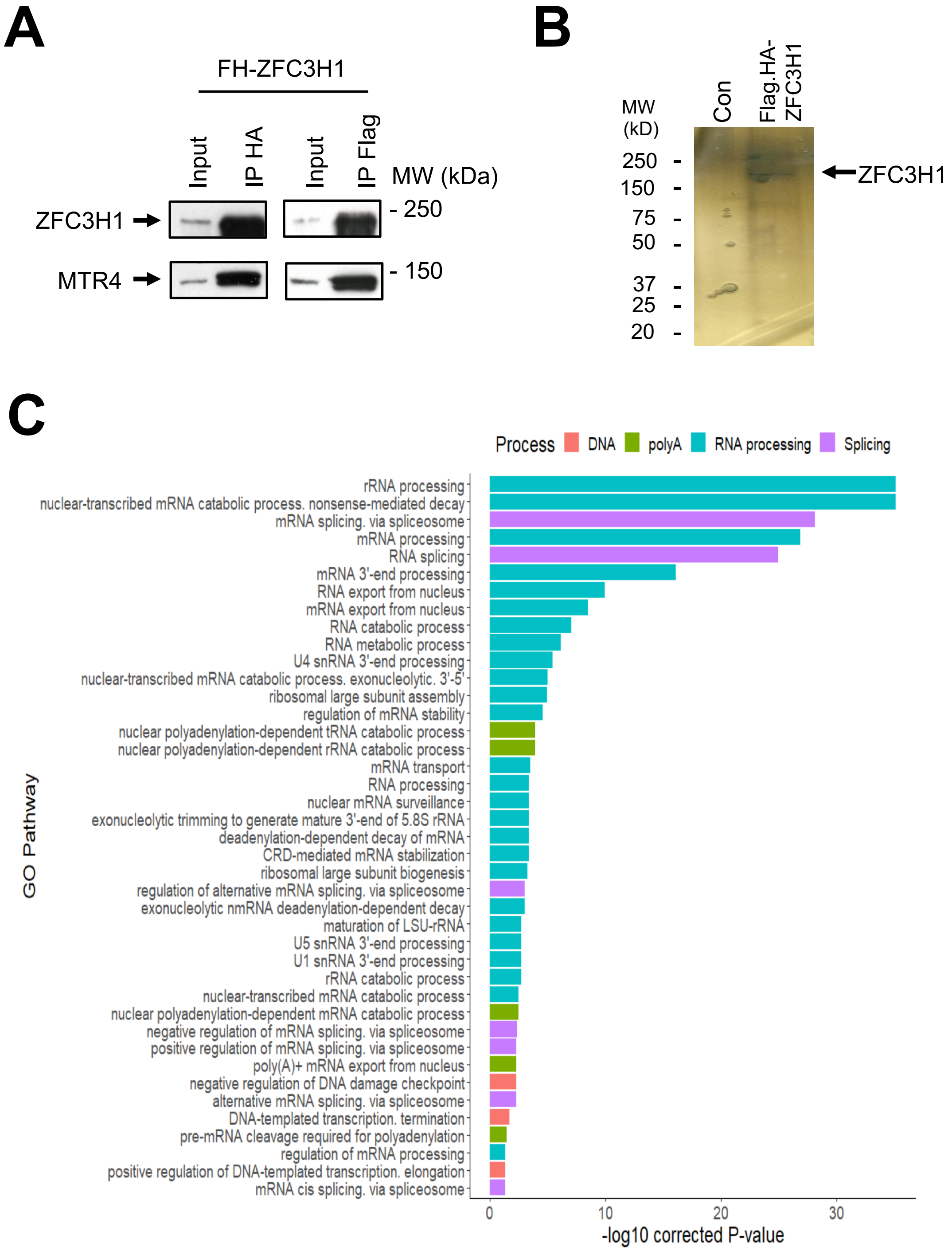

Supplementary Fig. 1

Supplementary Fig 1. Characterization of the endogenous ZFC3H1 complex. **A** Extracts of HEK-293T expressing Flag-HA-tagged ZFC3H1 (FH-ZFC3H1) were immunoprecipitated using anti-HA or anti-Flag antibodies, as indicated. Immunoprecipitates were analysed by immunoblotting using anti-ZFC3H1 or anti-MTR4 antibodies, as indicated. Molecular weight markers in kDa are shown at right. **B** SDS-PAGE analysis followed by silver staining of eluates of tandem affinity purified nuclear extracts from 293T stably expressing Flag-HA-ZFC3H1 or control cells. **C** Representative gene ontology pathways (biological pathways) of ZFC3H1-associated proteins identified by mass spectrometry (n=131; 2 or more peptides, FC>2). Source data are provided as a Source Data file.

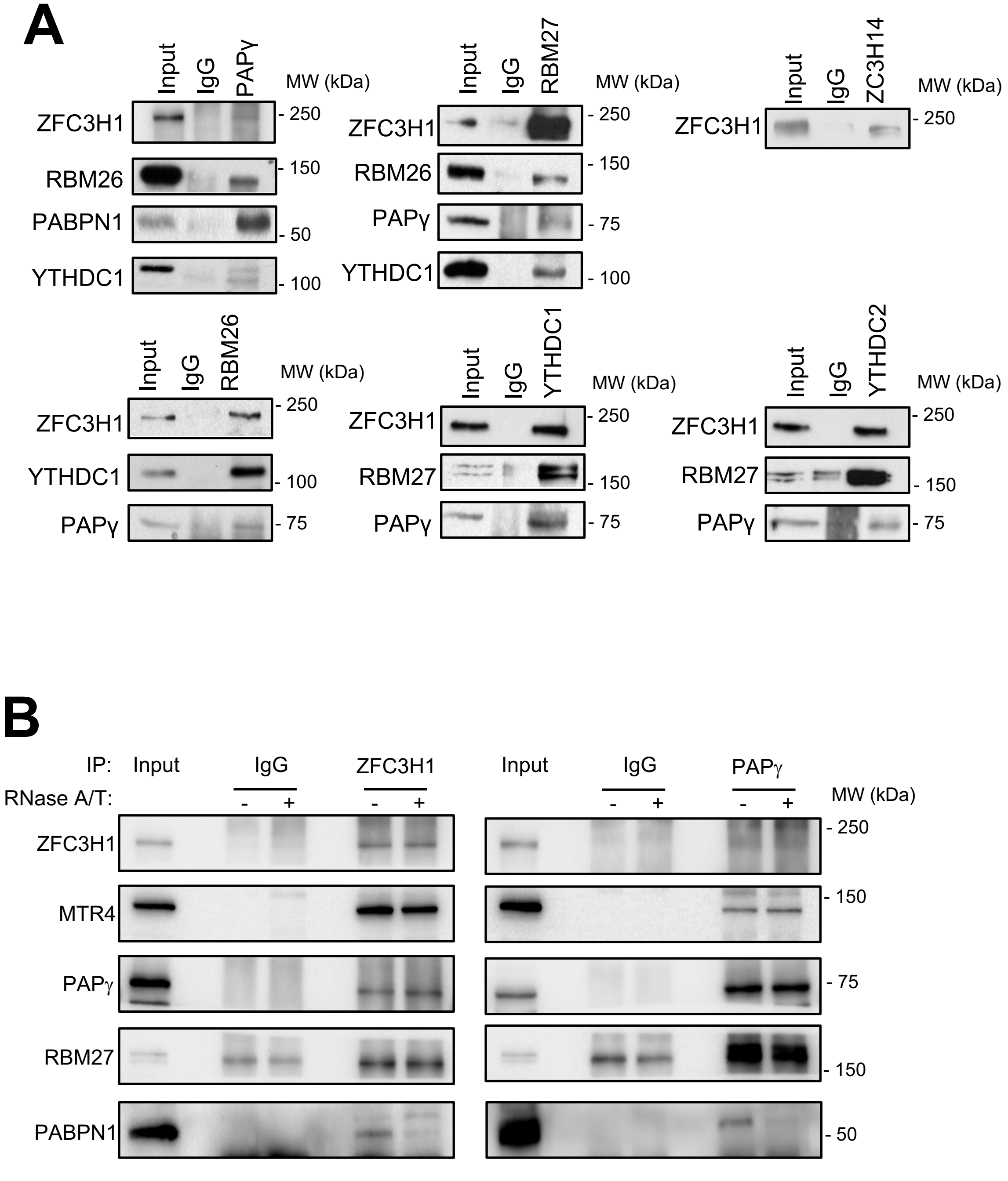

Supplementary Fig. 2

Supplementary Fig 2 Interactions between different PAXT subunits are mostly RNA-independent. A Co-immunoprecipitation analysis of ZFC3H1 interactome. HeLa nuclear extracts were immunoprecipitated using an IgG control or antibodies indicated on the figure above the blots. Immunoprecipitates and an aliquot of nuclear extract (input, 5%) were analyzed by SDS-PAGE followed by immunoblot using antibodies against ZFC3H1, RBM26, RBM27, YTHDC1, YTHDC2, PABPN1 or PAPγ, as indicated. Molecular weight markers in kDa are shown at right. **B** HeLa nuclear extracts were immunoprecipitated using antibodies against ZFC3H1, PAPγ or an IgG control and treated with RNase A/T or mock-treated, as indicated on the figure. Immunoprecipitates and an aliquot of nuclear extract (input, 5%) were analyzed by SDS-PAGE followed by immunoblot using the antibodies indicated on the right of the blots. Molecular weight markers in kDa are shown at right. Source data are provided as a Source Data file.

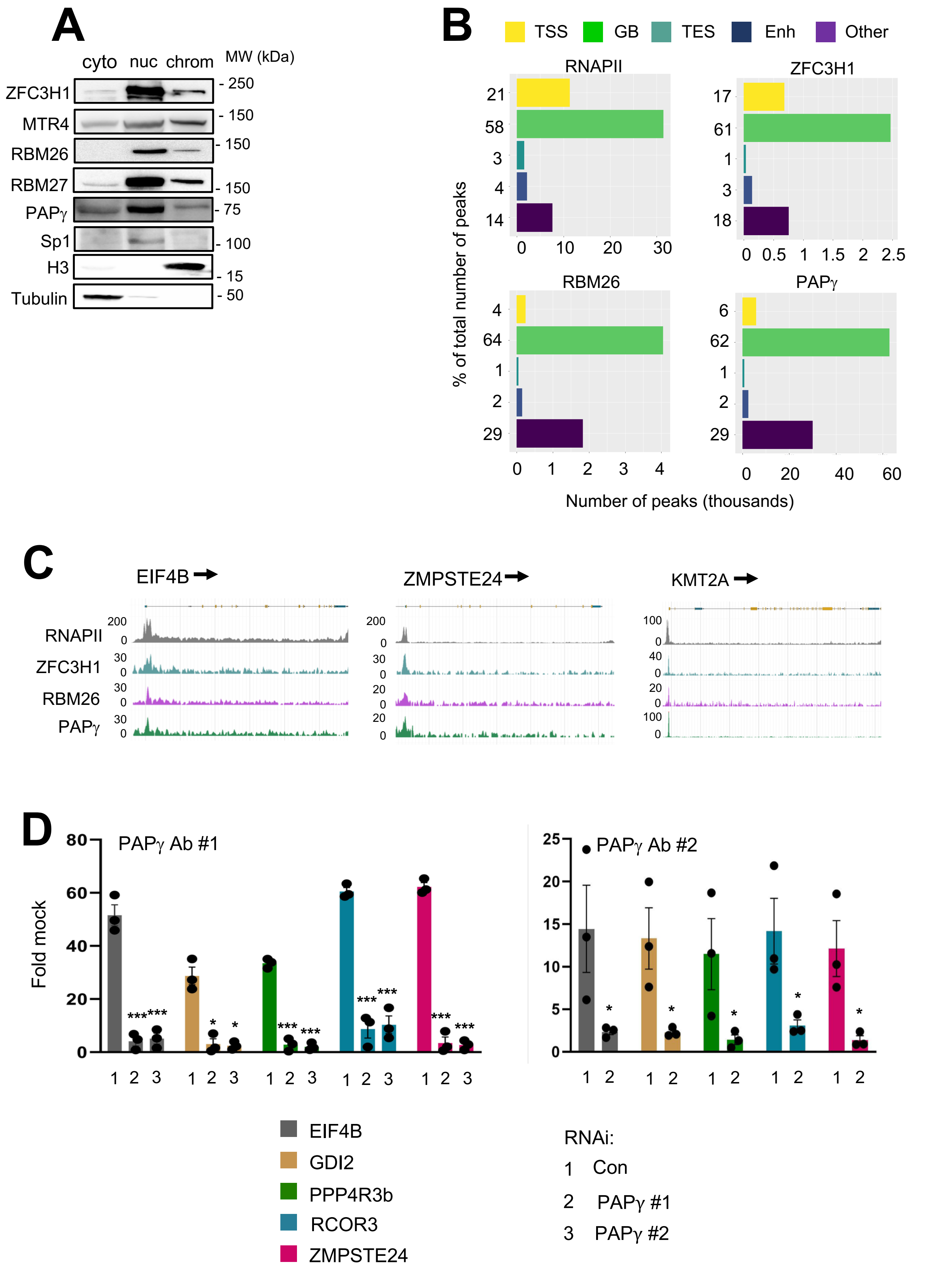

Supplementary Fig. 3

Supplementary Fig 3 PAXT subunits are associated with chromatin. A Cytosolic (cyto), nucleoplasmic (nuc) and chromatin (chrom) extracts of HeLa cells were analyzed by immunoblotting using the indicated antibodies. Molecular weight markers in kDa are shown at right. **B** Distribution of ChIP-seq peaks of RNAPII, ZFC3H1, RBM26 or PAPγ at transcription start site (TSS), gene body (GB), transcript end site (TES), enhancers (Enh) or other regions (Other). Number of peaks expressed as a percentage of the total peaks is shown at the left of the histograms, and total numbers of peaks are shown below. **C** Browser shots of RNAPII, ZFC3H1, RBM26, and PAPγ ChIP-seq signal over representative genes in HeLa cells. A schematic representation of the gene is shown above. **D** Anti-PAPγ specifically recognizes PAPγ. Occupancy of PAPγ at the TSS of the indicated genes detected by ChIP-qPCR using PAPγ antibodies (24284-1 and A302-426A, respectively) and chromatin from Hela cells transfected with siRNAs targeting PAPγ or a non-targeting control, as indicated. Data represent mean ± SEM obtained from 3 independent experiments (****P* < 0.001***P* < 0.01, **P* < 0.05, independent Student's *t* test). Source data are provided as a Source Data file.

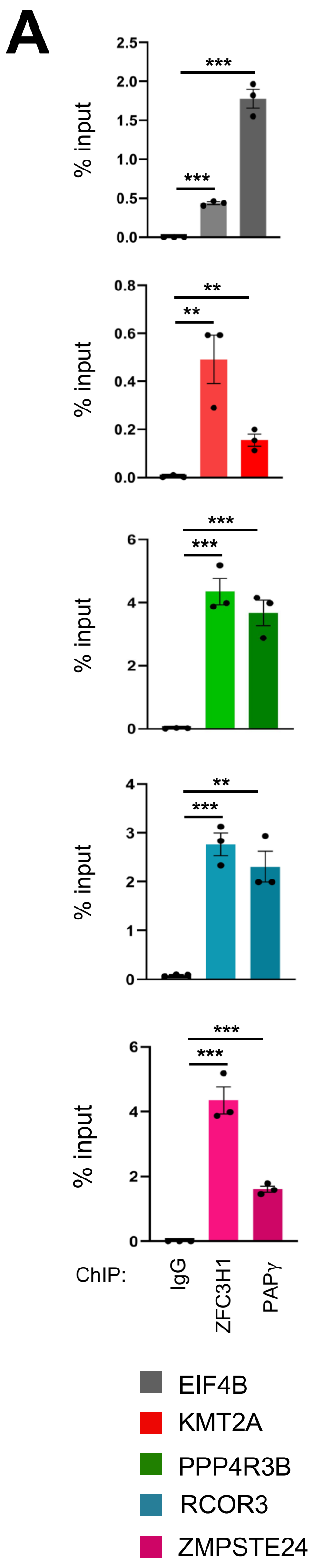

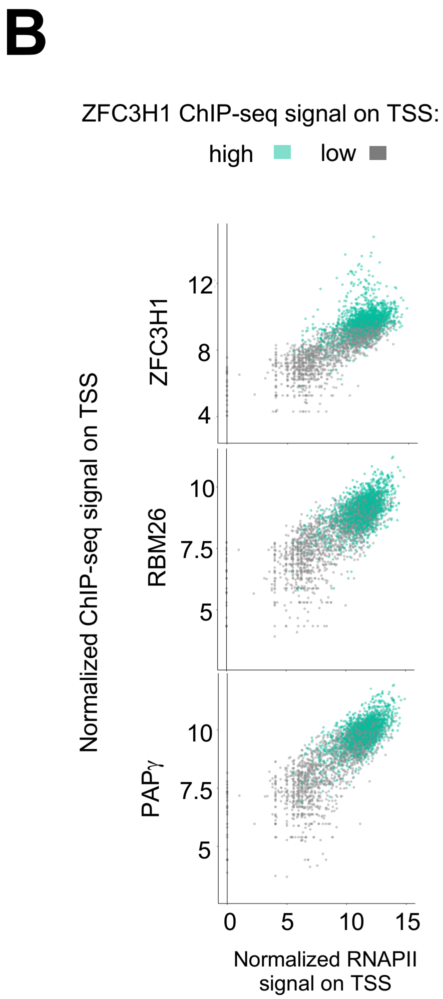

Supplementary Fig. 4

Supplementary Fig 4 PAXT subunits are co-recruited to chromatin. **A** ChIP-qPCR showing localization of ZFC3H1 and PAPγ at the TSS region of the indicated genes. Data represent mean ± SEM obtained from 3 independent experiments (****P* < 0.001, independent Student's *t* test). **B** Scatter plots showing the normalized ChIP-seq signal of ZFC3H1, RBM26 and PAPγ relative to that of RNAPII at the TSS of genes with a high or low occupancy of ZFC3H1 at the TSS, as indicated. Source data are provided as a Source Data file.

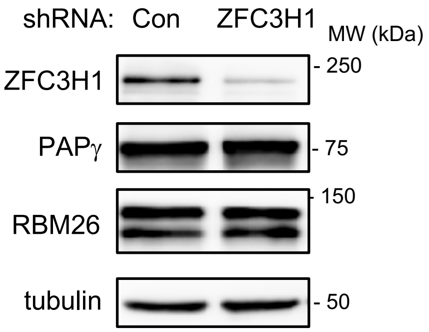

Supplementary Fig 5 Ablation of ZFC3H1 by specific shRNA. HeLa cells transduced with lentiviral particles expressing shRNA against ZFC3H1 or a non-targeting control were harvested 7 days post-infection. Extracts were analyzed by immunoblot using the indicated antibodies. Molecular weight markers in kDa are shown at right. Source data are provided as a Source Data file.

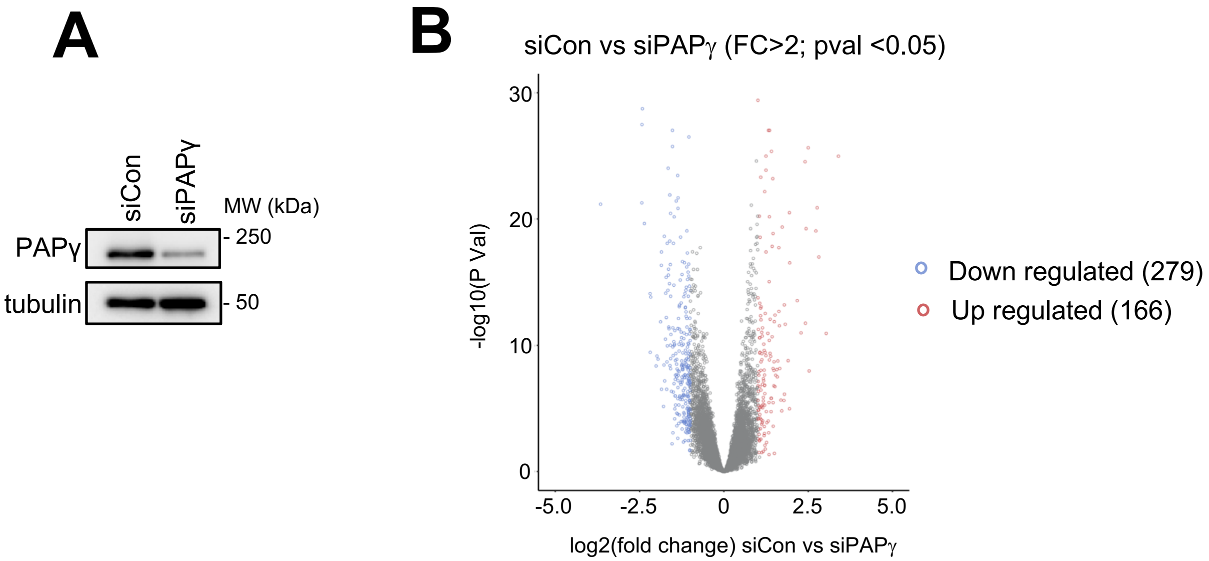

Supplementary Fig 6. Loss of PAPγ modestly affects the expression of mRNAs. A HeLa cells transfected with siRNA against PAPγ or a non-targeting control were harvested 4 days post-infection. Extracts were analyzed by immunoblot using the indicated antibodies. Molecular weight markers in kDa are shown at right. **B** Volcano plot showing the differential expression of mRNAs in extracts of HeLa cells transfected with siCon or siPAPγ. Source data are provided as a Source Data file.

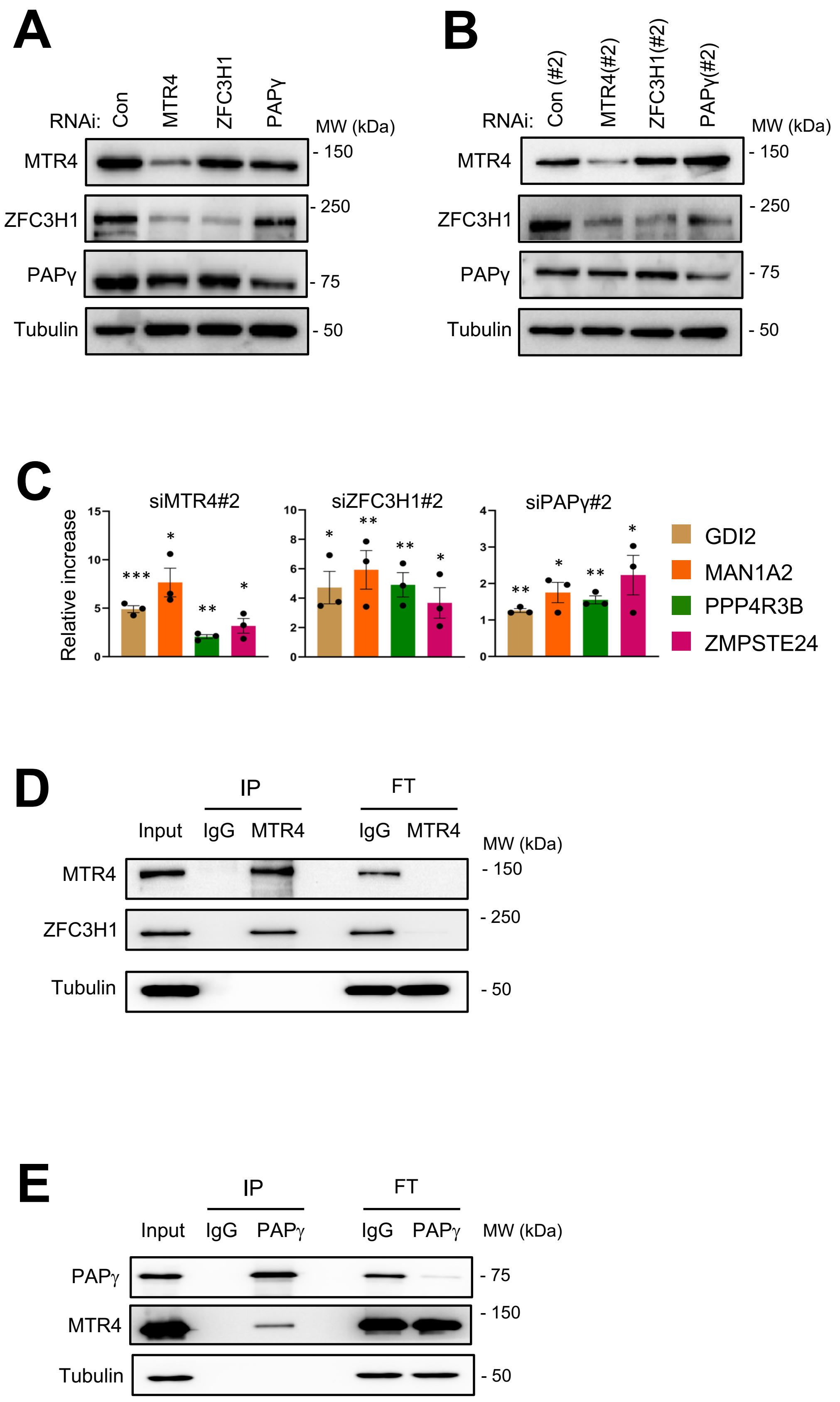

Supplementary Fig. 7

Supplementary Fig 7 Loss of PAXT subunits modulates the abundance of PROMPTs. A Immunoblot analysis of HeLa cell extracts following transfection with siRNAs directed against MTR4, ZFC3H1, PAPγ, or a control (con), as indicated on the figure. Molecular weight markers in kDa are shown at right. **B** Immunoblot analysis of HeLa cell extracts following transfection with siMTR4#2, siZFC3H1#2, siPAPγ#2, or a control (con#2), as indicated on the figure. Molecular weight markers in kDa are shown at right. **C** Total RNA extracts of HeLa cells transfected with the siRNAs shown in B were analyzed by RT-q-PCR using the oligonucleotide pairs indicated and the values were normalized to those for the control transfection, which was attributed a value of 1. Data represent mean ± SEM obtained from 3 independent experiments (***P < 0.001, **P < 0.01, *P < 0.05, independent Student's *t* test). **D, E** An aliquot of HeLa cell extract (input) and immunoprecipitates (IP) obtained using control IgG, anti-MTR4 (D) or anti-PAPγ (E) antibodies, as indicated, and flow-through samples (FT) were analyzed by Western blot using the indicated antibodies. Molecular weight markers in kDa are shown at right. Source data are provided as a Source Data file.

Table S1. Antibodies used in this study.

Target	Reference	Supplier	Dilution (immunoblot)
CPSF6	A301-356A	Bethyl Laboratories	1/1000
Flag	M2, F1804	Sigma-Aldrich	1/1000
HA	SC-7392	SCBT	1/1000
Histone H3	Ab1791	Abcam	1/5000
IgG mouse	SC-2025	SCBT	1/5000
IgG rabbit	SC-2027	SCBT	1/5000
MTR4 (IP)	ab70552	Abcam	-
MTR4 (WB)	A300-614A	Bethyl Laboratories	1/1000
MTR4 (RIP and WB)	12719-2-AP	Proteintech	1/1000
PABPN1	66807-1-Ig	Proteintech	1/1000
PAPγ (WB)	A302-427A	Bethyl Laboratories	1/1000
PAPγ (IP, WB, ChIP, ChIP-seq)	24284-1-AP	Proteintech	1/1000
PAPγ (IP, ChIP)	A302-426A	Bethyl Laboratories	-
RBM26 (IP, WB)	A301-215A	Bethyl Laboratories	1/1000
RBM27	A301-234A	Bethyl Laboratories	1/5000
RNAPII	F12; SC-55492	SCBT	-
Sp1	SC-59	SCBT	1/200
Tubulin	DM1A, T6199	Sigma-Aldrich	1/10000
YTHDC1	A305-096A	Bethyl Laboratories	1/5000
YTHDC2	A303-025A	Bethyl Laboratories	1/5000
ZC3H3	A301-878A	Bethyl Laboratories	1/5000
ZC3H14	A303-992A	Bethyl Laboratories	1/5000
ZFC3H1	A301-457A	Bethyl Laboratories	1/1000

Table S2. Oligodeoxynucleotide primers used in this study.

ssODN for genome editing	
Name	Sequence
ZFC3H1 -N ter	AATTGTCGGGCGGATCCCCGGACGGAGGGCTAAGGTTGTGTGGAAGGCGC TGCTCGCGAGATGGACTACAAAGACGATGACGACAAGCTCCATGGAGGATA CCCATACGATGTTCCAGATTACGCTGGAGGACTCGCGACCGCAGATACTCC GGCCCCGGCCTCCAGTGGCCTCTCGCCGAAGGAAGAAGGGGAG

Primers for RT-qPCR (PROMPTs)		
Name	Forward	Reverse
EIF4B	GGCGAAATGGATGTGCTGAA	CGCCTGTAATCCCAGCTACT
GDI2	GGTTTCATTGGGAAGCCGATC	GCTTCTGCTTACACCTGTGTC
KMT2A	AAACCTACGTCCCTCCACTG	GCGCTGTAACAACGGAATCT
MAN1A2	GAGCGCGAAAGAAGAGAAGG	AGCCATTAACTCCGGTGACA
MRPL20	TCTAGCGTGGGTGACAGATC	CATGCACTTGTGGTTCAGCT
PPP4R33	CCCATCCCCGCTATATTCGT	ACACTGACCCACGTTGTAGT
RCOR3	GGAGTGAGTGCTGTGGAGTG	ATAAACAGCCCCCAATGCCA
RPL8	GCCAAGTGGTTGAGTGACCT	CCTGCAAACCATGACGTTCC
SLC4A1	ACCTCTCCAACACAAGCTCA	CACAGCACTGCGATCTCTTC
ZMPSTE24	AGGAGGCTAGGAAGGACTGA	CGTCCACTCTCTCATACGCT

Primers for ChIP and Re-ChIP		
Name	Forward	Reverse
EIF4B	CTCTTTCCCTCTCCCAACAT	CGACTTCAGGAAATCAGTGG
GDI2	TCTGTGCCCTCCTTGTTTAG	AAACCTGGCTTTCACTTTCA
KMT2A	CGAGGCCGCTATACAGATTG	GTGAAGTGAAGCAGCGAGAG
PPP4R3b	GTTGGATGGCACCAGTATGT	CTTCCTTTCTGTCCCTGGTT
RCOR3	CAGAGAGGGCAAAGAGTCC	CCCAACCAAGCGCTAATAAA
ZMPSTE24	GTTGATGCAACACAGCTCAG	CAGACACACTCTTCCCCTTG

Primers for PAT assay		
Name	Forward	Reverse
PPP4R3B	AAAAGAACGTGGAGCCCTTT	GCCTGGAGAGTTGTGTCCAT
GDI2	GGTTTCATTGGGAAGCCGATC	GCTTCTGCTTACACCTGTGTC
ZMPSTE24	AGGAGGCTAGGAAGGACTGA	CGTCCACTCTCTCATACGCT
K-Ras	TGTATAGTGTAAACTGAAACA TGCAC	GTCACTGTAACTATTTTTATTAC

Table S3. Sequence targeted by siRNAs used in this study.

siRNAs	
**Target**	Sequence
**Control**	GCGCGCUUUGUAGGAUUCG(dTdT)
**Control #2**	UCUGCAAGGUUAGGCGUCU(dTdT)
**MTR4**	ACACUGAGCUGGAAAAUAA(dTdT)
**MTR4 #2**	AAGAAGUAUUCAGUAAUGC (dtdT)
**ZFC3H1**	CUAUAGCAGUCCAUCUUAA(dTdT)
**ZFC3H1 #2**	GUACAAUCGAUUCAUGAAA (dTdT)
**PAPγ**	ACAUCAAGAUGGCAUUAGA(dTdT)
**PAPγ #2**	UUUAUCUCAGGUUUCAAAG(dTdT)
