# Supplementary material for: PAPγ associates with PAXT nuclear exosome to control the abundance of PROMPT ncRNAs
Source: Nat Commun. 2023 Oct 24;14:6745. doi: 10.1038/s41467-023-42620-9 (PMC10598014; doi:10.1038/s41467-023-42620-9)
Supplement: Supplementary file 2 — Description of Additional Supplementary Files [file 41467_2023_42620_MOESM2_ESM.pdf]

Description of Additional Supplementary Files

Supplementary Data 1 : Proteins identified as interactants of ZFC3H1 detected by tandem affinity purification followed by tandem mass spectrometry.
