# Supplementary material for: PAPγ associates with PAXT nuclear exosome to control the abundance of PROMPT ncRNAs
Source: Nat Commun. 2023 Oct 24;14:6745. doi: 10.1038/s41467-023-42620-9 (PMC10598014; doi:10.1038/s41467-023-42620-9)
Supplement: Supplementary file 4 — Reporting Summary [file 41467_2023_42620_MOESM4_ESM.pdf]

nature portfolio

Corresponding author(s): Rosemary Kiernan

Last updated by author(s): 4/10/2023

Reporting Summary

Nature Portfolio wishes to improve the reproducibility of the work that we publish. This form provides structure for consistency and transparency in reporting. For further information on Nature Portfolio policies, see our Editorial Policies and the Editorial Policy Checklist.

Statistics

For all statistical analyses, confirm that the following items are present in the figure legend, table legend, main text, or Methods section.

n/a	Confirmed	
☐	☒	The exact sample size (*n*) for each experimental group/condition, given as a discrete number and unit of measurement
☐	☒	A statement on whether measurements were taken from distinct samples or whether the same sample was measured repeatedly
☐	☒	The statistical test(s) used AND whether they are one- or two-sided<br>*Only common tests should be described solely by name; describe more complex techniques in the Methods section.*
☒	☐	A description of all covariates tested
☒	☐	A description of any assumptions or corrections, such as tests of normality and adjustment for multiple comparisons
☐	☒	A full description of the statistical parameters including central tendency (e.g. means) or other basic estimates (e.g. regression coefficient) AND variation (e.g. standard deviation) or associated estimates of uncertainty (e.g. confidence intervals)
☒	☐	For null hypothesis testing, the test statistic (e.g. *F*, *t*, *r*) with confidence intervals, effect sizes, degrees of freedom and *P* value noted<br>*Give P values as exact values whenever suitable.*
☒	☐	For Bayesian analysis, information on the choice of priors and Markov chain Monte Carlo settings
☒	☐	For hierarchical and complex designs, identification of the appropriate level for tests and full reporting of outcomes
☐	☒	Estimates of effect sizes (e.g. Cohen's *d*, Pearson's *r*), indicating how they were calculated

Our web collection on statistics for biologists contains articles on many of the points above.

Software and code

Policy information about availability of computer code

Data collection	na
Data analysis	Published data analysis tools are referenced in the manuscript (refs 62 to 67). Opensource tools used were genomation (DOI: 10.18129/B9.bioc.genomation); 'Vennerable' R package (https://github.com/js229/Vennerable)

For manuscripts utilizing custom algorithms or software that are central to the research but not yet described in published literature, software must be made available to editors and reviewers. We strongly encourage code deposition in a community repository (e.g. GitHub). See the Nature Portfolio guidelines for submitting code & software for further information.

Data

Policy information about availability of data

All manuscripts must include a data availability statement. This statement should provide the following information, where applicable:

- Accession codes, unique identifiers, or web links for publicly available datasets
- A description of any restrictions on data availability
- For clinical datasets or third party data, please ensure that the statement adheres to our policy

GSE189157 and MSV00008942 are publicly available.

Research involving human participants, their data, or biological material

Policy information about studies with human participants or human data. See also policy information about sex, gender (identity/presentation), and sexual orientation and race, ethnicity and racism.

Reporting on sex and gender	NA
Reporting on race, ethnicity, or other socially relevant groupings	NA
Population characteristics	NA
Recruitment	NA
Ethics oversight	NA

Note that full information on the approval of the study protocol must also be provided in the manuscript.

Field-specific reporting

Please select the one below that is the best fit for your research. If you are not sure, read the appropriate sections before making your selection.

[x] Life sciences [ ] Behavioural & social sciences [ ] Ecological, evolutionary & environmental sciences

For a reference copy of the document with all sections, see nature.com/documents/nr-reporting-summary-flat.pdf

Life sciences study design

All studies must disclose on these points even when the disclosure is negative.

Sample size	sample size is indicated in the legend where appropriate
Data exclusions	no data exclusions
Replication	number of replicates is indicated in the legend where appropriate
Randomization	na
Blinding	na

Behavioural & social sciences study design

All studies must disclose on these points even when the disclosure is negative.

Study description	na
Research sample	na
Sampling strategy	na
Data collection	na
Timing	na
Data exclusions	na
Non-participation	na
Randomization	na

Ecological, evolutionary & environmental sciences study design

All studies must disclose on these points even when the disclosure is negative.

Study description	na
Research sample	na
Sampling strategy	na
Data collection	na
Timing and spatial scale	na
Data exclusions	na
Reproducibility	na
Randomization	na
Blinding	na

Did the study involve field work? ☐ Yes ☒ No

Field work, collection and transport

Field conditions	na
Location	na
Access & import/export	na
Disturbance	na

Reporting for specific materials, systems and methods

We require information from authors about some types of materials, experimental systems and methods used in many studies. Here, indicate whether each material, system or method listed is relevant to your study. If you are not sure if a list item applies to your research, read the appropriate section before selecting a response.

Materials & experimental systems

n/a	Involved in the study
☐	☒ Antibodies
☐	☒ Eukaryotic cell lines
☒	☐ Palaeontology and archaeology
☒	☐ Animals and other organisms
☒	☐ Clinical data
☒	☐ Dual use research of concern
☒	☐ Plants

Methods

n/a	Involved in the study
☐	☒ ChIP-seq
☒	☐ Flow cytometry
☒	☐ MRI-based neuroimaging

Antibodies

Antibodies used	CPSF6 A301-356A; Flag M2, F1804; HA SC-7392; Histone H3; IgG mouse; IgG rabbit; MTR4 (IP); MTR4 (WB); MTR4 (RIP and WB); PABPN1; PAPg (WB); PAPg (IP, ChIP, ChIP-seq); PAPg (IP, ChIP); RBM26 (IP, WB); RBM27; RNAPII; Sp1; Tubulin DM1A, T6199; YTHDC1; YTHDC2 A303-025A; ZC3H3 A301-878A; ZC3H14 A303-992A; ZFC3H1 A301-457A.
Validation	CPSF6 A301-356A https://www.fortislife.com/cms/files/A301-356A-3.pdf; Flag M2, F1804 https://www.sigmaaldrich.com/FR/en/sds/sigma/f1804<br>HA SC-7392 https://datasheets.scbt.com/sc-7392.pdf<br>Histone H3 https://www.abcam.com/products/primary-antibodies/histone-h3-antibody-nuclear-marker-and-chip-grade-ab1791.pdf

IgG mouse https://datasheets.scbt.com/sc-2025.pdf
IgG rabbit https://datasheets.scbt.com/sc-2027.pdf
MTR4 (IP) https://www.abcam.com/products/primary-antibodies/mtr4-antibody-ab70552.pdf
MTR4 (WB) https://www.fortislife.com/cms/files/A300-614A-4.pdf
MTR4 (RIP and WB) https://www.ptglab.com/products/pictures/pdf/12719-2-AP.pdf. Antibody was also validated by specific knock-down of the protein followed by immunoblot.
PABPN1 https://www.ptglab.com/products/pictures/pdf/66807-1-Ig.pdf
PAPg (WB) https://www.fortislife.com/cms/files/A302-427A-1.pdf
PAPg (IP, ChIP, ChIP-seq) https://www.ptglab.com/products/pictures/pdf/24284-1-AP.pdf. Antibody was also validated by specific knock-down of the protein followed by immunoblot or by ChIP-qPCR.
PAPg (IP, ChIP) https://www.fortislife.com/cms/files/A302-426A-1.pdf
RBM26 (IP, WB) https://www.fortislife.com/cms/files/A301-215A-1.pdf
RBM27 https://www.fortislife.com/cms/files/A301-234A-1.pdf
RNAPII https://datasheets.scbt.com/sc-55492.pdf

Sp1 https://datasheets.scbt.com/sc-59.pdf

Tubulin DM1A, T6199 Sigma-Aldrich https://www.sigmaaldrich.com/FR/en/sds/sigma/t6199

YTHDC1 https://www.fortislife.com/cms/files/A305-096A-1.pdf

YTHDC2 A303-025A https://www.fortislife.com/cms/files/A303-025A-1.pdf

ZC3H3 A301-878A https://www.fortislife.com/cms/files/A301-878A-1.pdf

ZC3H14 A303-992A https://www.fortislife.com/cms/files/A303-992A-1.pdf
ZFC3H1 A301-457A https://www.fortislife.com/cms/files/A301-457A-4.pdf. A301-457A was also validated by specific knock-down of the protein followed by immunoblot.

Eukaryotic cell lines

Policy information about cell lines and Sex and Gender in Research

Cell line source(s)	HeLa and 293T were obtained from ATCC
Authentication	cell lines were not authenticated
Mycoplasma contamination	All cell lines tested negative for Mycoplasma contamination (PCR and ELISA)
Commonly misidentified lines (See ICLAC register)	HeLa. It was used because it is a widely used cell line in molecular biology research.

Palaeontology and Archaeology

Specimen provenance	na
Specimen deposition	na
Dating methods	na

☐ Tick this box to confirm that the raw and calibrated dates are available in the paper or in Supplementary Information.

Ethics oversight	na

Note that full information on the approval of the study protocol must also be provided in the manuscript.

Animals and other research organisms

Policy information about studies involving animals; ARRIVE guidelines recommended for reporting animal research, and Sex and Gender in Research

Laboratory animals	na
Wild animals	na

Reporting on sex	na
Field-collected samples	na
Ethics oversight	na

Note that full information on the approval of the study protocol must also be provided in the manuscript.

Clinical data

Policy information about clinical studies
All manuscripts should comply with the ICMJE guidelines for publication of clinical research and a completed CONSORT checklist must be included with all submissions.

Clinical trial registration	na
Study protocol	na
Data collection	na
Outcomes	na

Dual use research of concern

Policy information about dual use research of concern

Hazards

Could the accidental, deliberate or reckless misuse of agents or technologies generated in the work, or the application of information presented in the manuscript, pose a threat to:

No	Yes	
☒	☐	Public health
☒	☐	National security
☒	☐	Crops and/or livestock
☒	☐	Ecosystems
☒	☐	Any other significant area

Experiments of concern

Does the work involve any of these experiments of concern:

No	Yes	
☒	☐	Demonstrate how to render a vaccine ineffective
☒	☐	Confer resistance to therapeutically useful antibiotics or antiviral agents
☒	☐	Enhance the virulence of a pathogen or render a nonpathogen virulent
☒	☐	Increase transmissibility of a pathogen
☒	☐	Alter the host range of a pathogen
☒	☐	Enable evasion of diagnostic/detection modalities
☒	☐	Enable the weaponization of a biological agent or toxin
☒	☐	Any other potentially harmful combination of experiments and agents

Plants

Seed stocks	na
Novel plant genotypes	na
Authentication	na

ChIP-seq

Data deposition

- [x] Confirm that both raw and final processed data have been deposited in a public database such as GEO.
- [ ] Confirm that you have deposited or provided access to graph files (e.g. BED files) for the called peaks.

Data access links<br>*May remain private before publication.*	The datasets generated during the current study are publicly available in the GEO (GSE189157)
Files in database submission	GSM5695517 Z1_shCtrl<br>GSM5695519 PAPgamma_shCtrl<br>GSM5695520 RBM26_shCtrl<br>GSM5695521 Z1_shZ1<br>GSM5695523 PAPgamma_shZ1<br>GSM5695524 RBM26_shZ1<br>GSM5695525 Input shCtrl<br>GSM5695526 Input_shZ1<br>GSM6213312 PolII rep1<br>GSM6213313 Z1 rep1<br>GSM6213314 Input_1<br>GSM6213315 PAPOLG<br>GSM6213316 RBM26<br>GSM6213317 Input_2<br>GSM6213318 Z1_shCtrl rep2<br>GSM6213319 Z1_shZ1 rep2<br>GSM6213320 Input3_shCtrl<br>GSM6213321 Input3_shZ1
Genome browser session<br>(e.g. UCSC)	available from GEO

Methodology

Replicates	biological replicates
Sequencing depth	read length is 75 bp, single end
Antibodies	antibodies used for ChIP-seq are indicated in supplementary Table S2 and on GEO
Peak calling parameters	peak calling was performed using NormR package (parameter FDR &e-4, 200 bp sliding windows). Number of peaks for FDR 1e-4 is given in supplentary Fig S2B
Data quality	Fastqc- quality control
Software	Alignment of reads was performed with BWA+samTools as described in the methods section 'bioinformatic analysis'. Normalization, bin-wide quantification and matrices extraction was performed with deepTools as described in the methods section 'bioinformatic analysis'

Flow Cytometry

Plots

Confirm that:

- [ ] The axis labels state the marker and fluorochrome used (e.g. CD4-FITC).
- [ ] The axis scales are clearly visible. Include numbers along axes only for bottom left plot of group (a 'group' is an analysis of identical markers).
- [ ] All plots are contour plots with outliers or pseudocolor plots.
- [ ] A numerical value for number of cells or percentage (with statistics) is provided.

Methodology

Sample preparation	*Describe the sample preparation, detailing the biological source of the cells and any tissue processing steps used.*
Instrument	*Identify the instrument used for data collection, specifying make and model number.*

Software | *Describe the software used to collect and analyze the flow cytometry data. For custom code that has been deposited into a community repository, provide accession details.*

Cell population abundance | *Describe the abundance of the relevant cell populations within post-sort fractions, providing details on the purity of the samples and how it was determined.*

Gating strategy | *Describe the gating strategy used for all relevant experiments, specifying the preliminary FSC/SSC gates of the starting cell population, indicating where boundaries between "positive" and "negative" staining cell populations are defined.*

☐ Tick this box to confirm that a figure exemplifying the gating strategy is provided in the Supplementary Information.

Magnetic resonance imaging

Experimental design

Design type | *Indicate task or resting state; event-related or block design.*

Design specifications | *Specify the number of blocks, trials or experimental units per session and/or subject, and specify the length of each trial or block (if trials are blocked) and interval between trials.*

Behavioral performance measures | *State number and/or type of variables recorded (e.g. correct button press, response time) and what statistics were used to establish that the subjects were performing the task as expected (e.g. mean, range, and/or standard deviation across subjects).*

Acquisition

Imaging type(s) | *Specify: functional, structural, diffusion, perfusion.*

Field strength | *Specify in Tesla*

Sequence & imaging parameters | *Specify the pulse sequence type (gradient echo, spin echo, etc.), imaging type (EPI, spiral, etc.), field of view, matrix size, slice thickness, orientation and TE/TR/flip angle.*

Area of acquisition | *State whether a whole brain scan was used OR define the area of acquisition, describing how the region was determined.*

Diffusion MRI ☐ Used ☐ Not used

Preprocessing

Preprocessing software | *Provide detail on software version and revision number and on specific parameters (model/functions, brain extraction, segmentation, smoothing kernel size, etc.).*

Normalization | *If data were normalized/standardized, describe the approach(es): specify linear or non-linear and define image types used for transformation OR indicate that data were not normalized and explain rationale for lack of normalization.*

Normalization template | *Describe the template used for normalization/transformation, specifying subject space or group standardized space (e.g. original Talairach, MNI305, ICBM152) OR indicate that the data were not normalized.*

Noise and artifact removal | *Describe your procedure(s) for artifact and structured noise removal, specifying motion parameters, tissue signals and physiological signals (heart rate, respiration).*

Volume censoring | *Define your software and/or method and criteria for volume censoring, and state the extent of such censoring.*

Statistical modeling & inference

Model type and settings | *Specify type (mass univariate, multivariate, RSA, predictive, etc.) and describe essential details of the model at the first and second levels (e.g. fixed, random or mixed effects; drift or auto-correlation).*

Effect(s) tested | *Define precise effect in terms of the task or stimulus conditions instead of psychological concepts and indicate whether ANOVA or factorial designs were used.*

Specify type of analysis: ☐ Whole brain ☐ ROI-based ☐ Both

Statistic type for inference | *Specify voxel-wise or cluster-wise and report all relevant parameters for cluster-wise methods.*

(See Eklund et al. 2016)

Correction | *Describe the type of correction and how it is obtained for multiple comparisons (e.g. FWE, FDR, permutation or Monte Carlo).*

Models & analysis

n/a	Involved in the study
☐	☐ Functional and/or effective connectivity
☐	☐ Graph analysis
☐	☐ Multivariate modeling or predictive analysis

Functional and/or effective connectivity	*Report the measures of dependence used and the model details (e.g. Pearson correlation, partial correlation, mutual information).*
Graph analysis	*Report the dependent variable and connectivity measure, specifying weighted graph or binarized graph, subject- or group-level, and the global and/or node summaries used (e.g. clustering coefficient, efficiency, etc.).*
Multivariate modeling and predictive analysis	*Specify independent variables, features extraction and dimension reduction, model, training and evaluation metrics.*
